# Straight Skeletons and Mitered Offsets of Nonconvex Polytopes

**DOI:** 10.1007/s00454-016-9811-5

**Published:** 2016-08-08

**Authors:** Franz Aurenhammer, Gernot Walzl

**Affiliations:** Institute for Theoretical Computer Science, University of Technology, Graz, Austria

**Keywords:** 3D straight skeleton, Mitered offset surface, Arrangement of planes, 68U05, 52B10

## Abstract

We give a concise definition of mitered offset surfaces for nonconvex polytopes in ${\mathbbm {R}}^3$, along with a proof of existence and a discussion of basic properties. These results imply the existence of 3D straight skeletons for general nonconvex polytopes. The geometric, topological, and algorithmic features of such skeletons are investigated, including a classification of their constructing events in the generic case. Our results extend to the weighted setting, to a larger class of polytope decompositions, and to general dimensions. For (weighted) straight skeletons of an *n*-facet polytope in ${\mathbbm {R}}^d$, an upper bound of $O(n^d)$ on their combinatorial complexity is derived. It relies on a novel layer partition for straight skeletons, and improves the trivial bound by an order of magnitude for $d \ge 3$.

## Introduction

Skeletal structures for geometric objects are an important concept in diverse areas of science. Motivated by applicational needs, different types of skeletons and their geometric and algorithmic properties have been studied, in computational geometry and also in more practically oriented fields.

The most prominent and widely used skeletal structure is the *medial axis*. It represents the set of centers of all multi-tangential circles (or spheres) inscribed to the object. Defined by distances to the boundary, the medial axis keeps a strong correspondence to the shape of the object; we refer the reader to [6, 8, 29] for an account of properties and further literature. However, even when the object boundary is piecewise linear (like for a polygon in the plane, or a polytope in 3-space), the medial axis contains curved elements when the object is nonconvex. This is a drawback in its computer construction and representation, and sometimes also in applications, especially in three dimensions where the medial axis attains a complex topology and a high algebraic degree. In fact, there have been several approaches to linearizing and simplifying this concept.

**Fig. 1:**
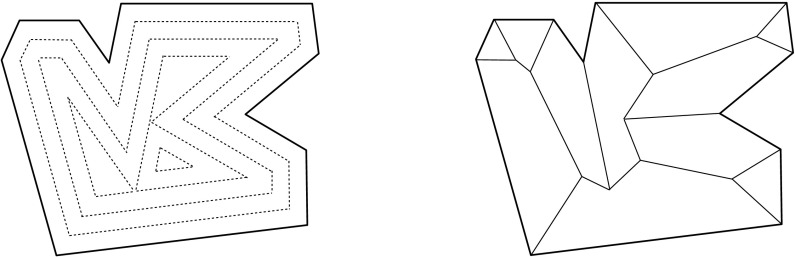
The boundary of the input polygon is translated inwards at unit speed (*left*). Thereby, the vertices of the polygon move on angle bisectors and trace out a unique tree structure—the straight skeleton (*right*). There are two kinds of ‘events’ that alter the polygon boundary combinatorially: A polygon edge shrinks to length zero (the *edge event*), or a polygon vertex runs into a non-incident polygon edge (the *split event*)

The so-called *straight skeleton* of a polygon (or polytope) offers a potential alternative. This structure is not defined via distances but rather in a procedural way, by means of a mitered boundary offsetting process which shrinks the object in a self-parallel way till it vanishes. In two dimensions, the shrinking process is combinatorially trivial, and the geometry of the straight skeleton is well understood [3, 4]. It is a unique graph whose leaf nodes are polygon vertices like with the medial axis, but whose arcs stem from angle bisectors and thus are straight-line segments. Figure 1 gives an illustration and some further explanations.

The planar straight skeleton has proved useful in various areas, including CAD (offset calculation and path generation), image processing (shape comparison and manipulation), architecture (automatic roof design), GIS (terrain and city modeling), and others; see e.g. [8, 16, 21, 32] and references therein. Still, designing fast and simple construction algorithms has remained a challenge, though powerful tools like motorcycle graphs [21] and gradient-based decompositions [16] have been developed, which are of interest in their own right.

It is desirable to find appropriate generalizations of the straight skeleton to three dimensions, as this would provide piecewise linear solutions for three main operations on nonconvex 3D polytopes which the medial axis cannot offer: *Offset calculation* (a standard operation in CAD), the construction of a *skeletal structure* (that encodes the shape of the polytope in a suitable manner), and *decomposition* (into a polyhedral mesh with simple cells). However, the precise definition of the shrinking process in 3D bears difficulties, and even the existence of a mitered offset surface which is continuous and not self-intersecting is no longer trivial.

Surprisingly, not much attention has been paid in computational geometry to these basic questions. Intuitively speaking, *shrinking* a given polytope means offsetting its boundary surface in inward direction, in a self-parallel way and at unit speed. Thereby the polytope facets ‘sweep out’ the cells of the 3D straight skeleton. The polytope edges (which move in angle bisector planes), and the polytope vertices (which move along trisector lines), trace out the sheets and the spokes of the skeleton, respectively, which border its cells. The shrinking polytope undergoes changes of various kinds, purely geometrical, or of combinatorial nature (changes in the boundary structure), or topological (appearance or merge of tunnels, or breaking apart).

Erickson (see [18]) observed the interesting fact that offset surfaces that arise in this way may be ambiguous, leaving different choices for the shrinking process and thus for the skeleton construction. Indeed, we encounter this problem already ‘in the first moment’, when polytope vertices of higher degree have to be resolved into several vertices with different incidence structure on the offset polytope surface: In general, only polytope vertices of degree 3 behave like polygon vertices, in the sense that they do not split when the object is shrunk infinitesimally. An elegant idea how to reduce the vertex resolution problem to a two-dimensional problem is proposed in Barequet et al. [12], using the combinatorial structure of the weighted^1^ straight skeleton [7, 13, 21] in a sectional plane that cuts off the polytope vertex in question. It remains unclear, though, how to generalize this method to vertices whose incident polytope faces positively span 3-space (for example, saddle point type vertices), as the use of more than one sectional plane leads to the necessity of merging planar straight skeletons, which is an unsolved problem.

The present paper, which contains and extends material from the conference papers [9, 10], is further concerned with these questions. Necessary and sufficient conditions for valid mitered offset surfaces of a nonconvex polytope are given (Sect. 3), along with a proof of existence, and a discussion of their basic properties and ambiguities. An (inevitably general) definition of nonconvex polytopes is stated in Sect. 2. We then revisit the two-dimensional reduction of the vertex resolution problem, and generalize it for polytope vertices of arbitrary type (Sect. 4). This solves, at the same time, the 3D straight skeleton construction problem for nonconvex polytopes (Sect. 5), as polytope vertices of higher degree caused by the shrinking process can be treated in the same uniform manner. In fact, for our vertex splitting algorithm, the type of a skeleton construction event needs not be known in order to process the event correctly. We gain further insight into the structure of 3D straight skeletons, concerning their facial structure and topology in Sect. 5, and their geometry in Sect. 6, where we also provide an enumeration and categorization of all events that can take place in the generic case. A more general class of polytope decompositions is introduced in Sect. 7, where certain members can be computed by a simple direct method. The basic concepts and proofs in Sects. 3–7 do not depend on the offset speed, nor on the dimension, which allows for extending our results to the weighted setting (Sect. 8), and to general dimensions (Sect. 9). We conclude the paper with some experimental results obtained by implementing our 3D straight skeleton construction algorithm (Sect. 10). Some potential applications are mentioned, like flattening a polytope [19], decomposing a polytope [22] into small monotone cells, and offsetting a polygonal mesh for the purpose of $\varepsilon$-thinning [34].

There are certain special cases where 3D straight skeleton algorithms have been known. First of all for *convex* polytopes, where the straight skeleton coincides with the medial axis, and can be interpreted as the (projected) lower envelope of *n* hyperplanes in 4-space that correspond to the *n* polytope facets. The skeleton therefore consists of convex cells whose overall size (combinatorial complexity) is $\Theta (n^2)$ in the worst case, and it can be computed in $O(n^2)$ time by any optimal 4D convex hull algorithm; see e.g. [27]. Similarly, the straight skeleton for *axis-aligned* (or orthogonal) polytopes is the medial axis in the $L_\infty$-metric, and has a quadratic behavior in size as well; the computation time increases by a polylogarithmic factor [12, 25]. In these particular settings, the straight skeleton is a unique structure. No algorithmic results or nontrivial upper size bounds have been known for (more) general polytopes. However, a super-quadratic lower bound of $\Omega (n^2 \alpha ^2(n))$ on the skeleton size exists [12].

We give an $O(n^d)$ upper bound on the combinatorial complexity of straight skeletons for arbitrary boundary-connected polytopes in *d*-space, including their positively weighted versions. This improves the trivial bound by an order of magnitude. The argument is based on a unique layer partition for straight skeletons and related cell complexes, introduced in Sect. 7, which may be of interest on its own.

## Polytope

Here we define the type of polytope we would like to work with, and give some related definitions and explanations.

A *convex polytope* is the finite intersection of closed halfspaces of Euclidean three-space ${\mathbbm {R}}^3$, with nonempty interior. A *polytope* is a bounded subset of ${\mathbbm {R}}^3$ which can be expressed as the finite union of convex polytopes. This definition is quite general. A polytope can have tunnels, voids, or even be disconnected. The boundary of a polytope has a facial structure and consists of *vertices* (points of intersection of three linearly independent supporting planes), *edges* (minimal closed^2^ subsets of intersection lines bounded by two vertices), and *facets* (minimal closed subsets of supporting planes bounded by edges). Note that polytope facets are connected, but are not required to be simply connected. Moreover, there might exist so-called *touching faces*, for example, a vertex or an edge touching the interior of a non-incident facet. That is, the boundary complex of a polytope needs not be face-to-face. Polytopes with boundary singularities of various kinds arise naturally during the shrinking process, even for the simple setting where the input polytope is homeomorphic to a ball and all its facets are triangles.

**Fig. 2:**
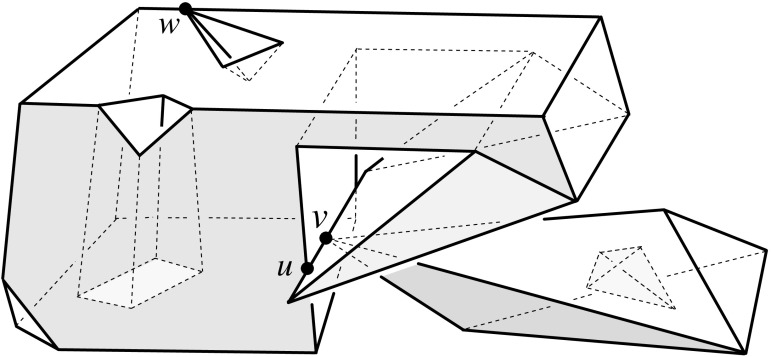
A polytope ${\mathcal{Q}}$ with complex topology. ${\mathcal{Q}}$ contains a tunnel, a void, and three mutually touching faces—a facet, an edge, and the vertex *v*. The bottommost facet is not simply connected. ${\mathcal{Q}}$ is a connected polytope, but is neither interior-connected nor boundary-connected. The closed complement $\overline{{\mathcal{Q}}}$ of ${\mathcal{Q}}$ (in a suitable bounding volume) is a valid polytope as well. When $\overline{{\mathcal{Q}}}$ is shrunk, i.e., when ${\mathcal{Q}}$ is expanded, a complicated combinatorial change on the boundary takes place at vertex *v*

A polytope, ${\mathcal{Q}}$, can have various types of vertices. It will turn out useful to draw the following general distinction: A vertex *v* of ${\mathcal{Q}}$ is called a *touching vertex* if there exists some $\varepsilon > 0$ such that each sphere, centered at *v* and having a positive radius of at most $\varepsilon$, intersects the boundary of ${\mathcal{Q}}$ in a disconnected set. (In the polytope in Fig. 2, the vertex *v* is of the touching type, but the vertices *u* and *w* are not.) A vertex is called *non-touching*, otherwise. Among the latter vertices, certain types are particularly relevant. A vertex *v* is *pointed* if there exists an open (geometric) disk whose intersection with ${\mathcal{Q}}$ is exactly *v*. A *saddle vertex* is incident to edges that positively span 3-space.^3^ These two types are exclusive, but not exhaustive among the non-touching vertices. If not already present in ${\mathcal{Q}}$’s boundary, touching vertices and saddle vertices will be created generically in the polytope offsetting process, including such having coplanar facets, or some facet with a reflex angle.

An edge *e* of a polytope ${\mathcal{Q}}$ is called *convex*, *reflex*, or *flat* respectively, if the dihedral interior angle spanned by *e*’s incident facets is smaller, larger, or equal to $\pi$. If *e* is part of a boundary singularity of ${\mathcal{Q}}$, then the definition is meant locally for each respective part of ${\mathcal{Q}}$. For instance, the edge $\overline{uv}$ in Fig. 2 is flat in the left part of the polytope, and convex in the right part. A non-touching vertex *v* of ${\mathcal{Q}}$ is called *convex* if all edges incident to *v* are convex. Likewise, *v* is called *reflex* if all its edges are reflex. Note that convex vertices are pointed, but reflex vertices are neither pointed nor saddle vertices. A saddle vertex necessarily has edges of both convexity types. The *degree* of a vertex *v* of ${\mathcal{Q}}$ is the number of edges of ${\mathcal{Q}}$ that are incident to *v*. Vertex degrees have to be at least 3, because vertices come from intersecting three or more planes.

We impose no general position assumption on a polytope, nor is this needed for our structural and algorithmic results. For the ease of exposition, a generic behavior of the facet offset planes will be assumed at certain places, though only temporarily.

## Valid Offset Surfaces

In this section, we give a characterizing definition of mitered offset surfaces and a proof of their existence, along with a discussion of some of their relevant properties.

### Characterization

Let ${\mathcal{Q}}$ be a polytope as defined in Sect. 2. Consider some vertex, *v*, of ${\mathcal{Q}}$ with degree *m*. We have $m \ge 3$, but the degree of *v* can be arbitrarily large, $m \le n-1$, where *n* counts the number of facets of ${\mathcal{Q}}$. To ease the subsequent description we assume, here and in Sect. 3.2, that *v* is a non-touching vertex; we will come back to the general situation (which is similar) in Sect. 4.2.

Let $f_1, \ldots , f_m$ be the facets of ${\mathcal{Q}}$ incident to *v*. Each such facet $f_i$ defines a supporting plane $H_i$, and we denote with $H^{\Delta }_i$ the parallel offset of $H_i$ by some fixed value ${\Delta > 0}$, inward with respect to ${\mathcal{Q}}$. Note that we may have $H^{\Delta }_i = H^{\Delta }_j$ for $i \ne j$, when two facets $f_i$ and $f_j$ are coplanar. Our interest is in the *arrangement* defined by the offset planes $H^{\Delta }_1, \ldots , H^{\Delta }_m$, that is, in the dissection of ${\mathbbm {R}}^3$ induced by these planes. This structure has to comprise all possible offset surfaces that may result (locally) from resolving the vertex *v*. We denote this arrangement by ${\mathcal{A}}(v)$; its combinatorial properties do not depend on $\Delta$, provided this offset parameter is positive.

Now center a sphere, *U*, at the vertex *v*, sufficiently small to intersect only faces incident to *v*. The intersection of *U* with the polytope ${\mathcal{Q}}$ is a spherical polygon, ${\mathcal{S}}$, which is simply connected because *v* is a non-touching vertex. Note that ${\mathcal{S}}$ is not necessarily contained in a hemisphere of *U* (for example, when *v* is a saddle vertex). In the following definition, the planes $H^{\Delta }_i$ serve as functions over the spherical domain ${\mathcal{S}}$. More precisely, $H^{\Delta }_i(x)$ measures the distance from *v* to $H^{\Delta }_i$ in the direction $x \in {\mathcal{S}}$.

#### Definition 3.1

A *valid offset surface* for *v* is (the graph of) a radial function $\Sigma$ over ${\mathcal{S}}$ that satisfies the following three conditions.

1. For every $x \in {\mathcal{S}}$ we have $\Sigma (x) = H^{\Delta }_i(x)$ for some index *i*.
2. $\Sigma (x) = \infty$ holds for all *x* on the boundary of ${\mathcal{S}}$.
3. $$\Sigma $$ is continuous.

A valid offset surface $\Sigma$ for *v* thus is a radially visible^4^ polyhedral terrain over ${\mathcal{S}}$, expressible as the union of certain facets from the arrangement ${\mathcal{A}}(v)$, by conditions (1) and (3), and locally fitting with its unbounded facets to the offset polytope surface by condition (2). In particular, $\Sigma$ has no self-intersections because $\Sigma$ is a radial function. Observe that $\Sigma$ in the limit $\Delta \rightarrow 0$ supports the facets of ${\mathcal{Q}}$ incident to *v*, because then all planes $H^{\Delta }_i$ concur at *v*. These properties are sufficient to have an offset polytope, ${\mathcal{Q}}^{\Delta }$, of ${\mathcal{Q}}$ defined after splitting the vertex *v* according to $\Sigma$. They are also necessary, which is evident except for radial visibility that we give a closer look now. (The existence of ${\mathcal{Q}}^{\Delta }$ is guaranteed by Theorem 3.3 in the next subsection.)

#### Lemma 3.2

Let $\Sigma '$ be the union of all facets of ${\mathcal{Q}}^{\Delta }$ that have changed combinatorially, as a result of resolving the vertex *v* of ${\mathcal{Q}}$. If $\Sigma '$ is not radially visible from *v*, then ${\mathcal{Q}}^{\Delta }$ contains some facet offsetting toward the *exterior* of ${\mathcal{Q}}^{\Delta }$. The converse of the statement holds, too.

#### Proof

Let *r* be an infinite ray originating at *v* and with $r \cap \Sigma ' \ne \emptyset$. The ray *r* can intersect $\Sigma '$ only transversely, because the offset planes $H^{\Delta }_i$ avoid *v*. Let $p_1$ be the first point of intersection, at facet $f^\Delta _1$, say. (Without loss of generality, *r* does not intersect any edge of $\Sigma '$. We can alter *r* infinitesimally, otherwise.) For increasing $\Delta$, the planes $H^{\Delta }_i$ move away from *v*, such that the facet $f^\Delta _1$ offsets toward the interior of ${\mathcal{Q}}^{\Delta }$. Now, if $\Sigma '$ is not radially visible with respect to *v*, then (and only then) a second point, $p_2$, of intersection of *r* with $\Sigma '$ exists such that the line segment $\overline{p_1p_2}$ lies inside ${\mathcal{Q}}^{\Delta }$. But the facet touched by $\overline{p_1p_2}$ at $p_2$ will offset toward the exterior of ${\mathcal{Q}}^{\Delta }$. The lemma follows. $\square$

Facets that shift in the ‘wrong’ direction contradict the polytope shrinking process, in the sense that a sequence of polytopes ${\mathcal{Q}}^{\Delta }$, obtained when increasing the offset parameter $\Delta$, is not ordered by containment. In particular, there will exist points inside ${\mathcal{Q}}$ which are swept over by the offsetting polytope boundary more than once. This fact is intolerable for the construction of a 3D straight skeleton, if the skeleton cells are supposed to partition the polytope ${\mathcal{Q}}$.

There exist surfaces for *v* which are not radially visible but do *not* self-intersect; see Fig. 4(middle). This shows that ruling out self-intersections is a condition too weak for our purposes. On the other hand, Definition 3.1 offers maximal generality. It conforms with the classical mitered offsetting process for polygons in the literature, but enables additional offset combinatorics in certain situations. The interested reader may consult Sect. 8.1 at this point, for a brief discussion of the planar case.

### Existence and Basic Properties

The question of the existence of valid offset surfaces arises. An affirmative answer follows from the results in [9, 10] described in Sect. 4.2. We find it instructive to give an alternative (and dimension-independent) proof, which is directly based on the offset plane arrangement ${\mathcal{A}}(v)$.

#### Theorem 3.3

A valid offset surface $\Sigma$ as in Definition 3.1 always exists. Moreover, $\Sigma$ can be chosen such that all its facets are unbounded.

#### Proof

Let ${\mathcal{K}}$ be the set of all unbounded cells of ${\mathcal{A}}(v)$ whose radial projection to the sphere *U* lies in the function domain ${\mathcal{S}}$. We claim that the boundary surface, $F({\mathcal{K}})$, that results from the union of these cells constitutes a valid offset surface. Clearly, $F({\mathcal{K}})$ is continuous and it satisfies condition (2). It remains to show that $F({\mathcal{K}})$ is radially visible from *v*. Assume that some ray *r* emanating from *v* intersects the boundary of a (convex) cell *C* in ${\mathcal{K}}$ a second time. Then *C* has to be adjacent there to another cell in ${\mathcal{K}}$; otherwise, by the continuity of $F({\mathcal{K}})$, some offset plane $H^{\Delta }_i$ contributing to $F({\mathcal{K}})$ would split *C* into a bounded and an unbounded part—a contradiction.

To get rid of the bounded facets of $F({\mathcal{K}})$ (if any), we now include into ${\mathcal{K}}$, one by one, cells adjacent to such facets. (These cells are all bounded; see Fig. 4 for an example of the cell adding process.) This process terminates, because the added cells will enlarge the unbounded facets of $F({\mathcal{K}})$ to such an extent that no bounded facets remain: For each unbounded facet of $F({\mathcal{K}})$, $f^{\Delta }_i$, which is extendable in this way, eventually some cells will be added that contain a facet from the same supporting plane $H^{\Delta }_i$. Upon termination, $F({\mathcal{K}})$ is a valid surface, as unbounded facets always offset in the ‘right’ direction and thus do not violate radial visibility by Lemma 3.2. $\square$

**Fig. 3:**
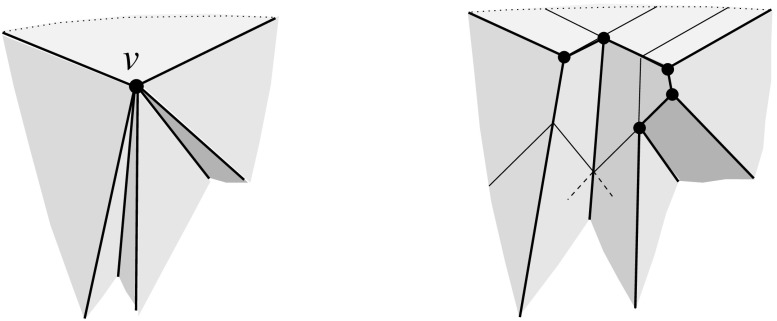
A polytope vertex *v* of degree 7 (*left*), and the surface of the union of all unbounded offset arrangement cells that radially project to the domain ${\mathcal{S}}$ (*right*)

**Fig. 4:**
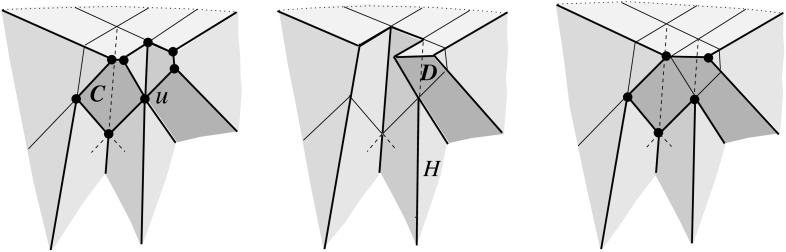
Adding the cell *C* (*left*), or the cell *D* (*middle*), or both cells (*right*)

Theorem 3.3 can be generalized to touching vertices (see Sect. 4.2) and is of fundamental importance for our considerations. The existence of a mitered offset boundary—and with it, the existence of a 3D straight skeleton—for general nonconvex polytopes in ${\mathbbm {R}}^3$ hinge upon its validity.

Valid offset surfaces are not unique in general; see [18]. The smallest possible example of ambiguity is for a saddle vertex of degree 4 whose edges alternate between being convex and reflex [9], as in Fig. 12. We use a bigger example to illustrate how valid offset surfaces are obtained from the offset plane arrangement. Consider the degree-7 vertex in Fig. 3(left). The displayed set of arrangement cells (right) is the smallest set that yields a valid offset surface. All facets of this surface are, by accident, unbounded. Still, we can add bounded cells while keeping the surface radially visible, for instance, the cell *C* shown in Fig. 4(left). This creates a surface vertex, *u*, of degree 6. Adding the cell *D* instead of *C* leads to a temporary violation of radial visibility (middle). We have the peculiar situation that one offset plane (marked with *H*) supports the surface with both sides now, such that one of the two facets defined by *H* (the hidden triangular facet of *D*) would shift toward the *exterior* of the polytope; cf. Lemma 3.2. This can be remedied by adding the cell *C* next (right). In the resulting valid surface, all facets are unbounded again. No more cells can be added in this example without destroying radial visibility beyond repair.

In conclusion, three valid solutions exist. In one of them, the vertex *v* gets resolved into 8 surface vertices, rather than only 5 as in the other two cases. (Such vertices are marked with ‘$\bullet$’ in the figures.) Moreover, a degree-6 vertex *u* occurs there.

These phenomena may be unwanted in applications. Higher-degree surface vertices complicate the resolution problem for future vertices that arise in the offsetting process for the polytope ${\mathcal{Q}}$, in a structural respect and also algorithmically when the resulting combinatorial changes (later called events) have to be implemented. Generally, offset surfaces of small combinatorial complexity seem desirable, also in view of a 3D straight skeleton construction for ${\mathcal{Q}}$. Bounded surface facets (we term them *orphan facets*) trace out extraneous skeleton cells, such that the volume swept over by an individual offset plane is not interior-connected any more; see Sect. 5. For this reason, we may require that each offset plane defines a single (and then unbounded) facet in the surface, as it is the case when a *convex* vertex *v* of ${\mathcal{Q}}$ is resolved: The unique surface then is the radial lower envelope^5^ of the offset planes, and *v* splits into vertices of degree 3 in the generic case, i.e., when no 4 offset planes pass through the same point. Indeed, we can have similar positive features for arbitrary polytope vertices *v*. (The case of touching vertices is covered in Corollary 4.4, see Sect. 4.2.)

#### Corollary 3.4

Let *v* be a non-touching vertex of the polytope ${\mathcal{Q}}$, and let *m* be the degree of *v*. There exists a valid (orphan-free) offset surface for *v* whose edge graph is a forest with at most $m-2$ inner vertices. In the generic case, all these vertices have degree 3.

#### Proof

By Theorem 3.3, there exists a valid offset surface $\Sigma$ for *v* such that all facets of $\Sigma$ are unbounded. The edge graph of $\Sigma$ then has no cycles, and is a tree or a forest if certain edges flatten out due to coplanarity of *v*’s polytope facets. To see that $\Sigma$ exclusively contains vertices of degree 3 in the generic case, we observe that surface vertices of degree ${\ge }$4 then are necessarily incident to some orphan facet: Let *w* have degree ${\ge }$4. Then *w* is incident to at least 4 facets and, because exactly 3 planes in ${\mathcal{A}}(v)$ pass through *w*, at least 2 facets for *w* stem from the same plane, say $H^{\Delta }_i$. But $H^{\Delta }_i$ cannot define 2 *unbounded* surface facets incident to *w* (by Definition 3.1 (2); points *x* with $\Sigma (x) = - \infty$ would exist, otherwise), so at least one of them is an orphan facet. The number of vertices of $\Sigma$ is at most $m-2$, the maximal number of inner nodes in a tree with *m* leaves. $\square$

Even under the restrictions in Corollary 3.4, the offset surface is not unique, as Figs. 3(right) and 4(right) show. We remark at this point that there exist valid offset surfaces where all vertices are of degree 3, but orphan facets are still present; see Fig. 8 in Sect. 4.2. The reason is that facets which do *not* share a vertex can arise from the same offset plane. With this observation, the degree argument in the proof of Corollary 3.4 implies:

#### Lemma 3.5

Let *v* be a vertex of ${\mathcal{Q}}$ (of arbitrary type), and let $\Sigma$ be some valid offset surface for *v*. If $\Sigma$ is orphan-free then all its vertices are of degree 3 in the generic case. The converse is not true, in general.

Let us observe that the lower (or upper) envelope of two valid offset surfaces for *v* is a valid offset surface as well. This directly follows from Definition 3.1. In other words, the set $\mathcal{X}$ of valid offset surfaces for *v* is closed under taking envelopes. This implies two partial orders on the elements in $\mathcal{X}$, and the existence of two unique *extreme surfaces* $\Sigma ^- , \Sigma ^+ \in \mathcal{X}$. Extreme surfaces are not necessarily orphan-free, as Fig. 8(middle) indicates: The maximum set of cells has been added to obtain $\Sigma ^+$.

The shrinking process for the polytope ${\mathcal{Q}}$ refers to the *inner offset* of its boundary. In fact, the quest for an *outer offset* for ${\mathcal{Q}}$, which may be relevant in certain practical applications (and where we have $\Delta < 0$ for the offset planes $H^{\Delta }_i$) leads to an equivalent problem: It can be viewed as an inner offset problem, when ${\mathcal{Q}}$ is replaced by the polytope $\overline{{\mathcal{Q}}}$ that results from taking the (closed) complement of ${\mathcal{Q}}$ in a suitable enclosing box. As a consequence, all our results are applicable to outer offsets as well.

## Reduction to Two Dimensions

For algorithmic purposes, it is of advantage to reduce the vertex resolution problem to one dimension less. We will distinguish two cases, depending on the type of the polytope vertex considered. More specifically, we discuss a two-dimensional reduction for pointed polytope vertices first, and then proceed to a generalization for arbitrary vertices, including the touching vertex type.

### Pointed Case Revisited

Let us consider any pointed vertex *v* of the polytope ${\mathcal{Q}}$. Recall from Sect. 2 that *v* needs not be a convex vertex, that is, *v* can have reflex incident edges. However, there exists a plane *E* that intersects all edges of ${\mathcal{Q}}$ incident to *v*. Moreover, as *v* is a non-touching vertex by assumption, *E* intersects ${\mathcal{Q}}$ (locally at *v*) in a simple polygon, which will be denoted by ${\mathcal{P}}$ in the sequel. If *v* is of degree *m* then ${\mathcal{P}}$ has *m* edges, $e_1, \ldots , e_m$.

When the offset parameter ${\Delta }$ increases in the shrinking process for ${\mathcal{Q}}$, the polygon ${\mathcal{P}}$ shrinks to the inside as well. More precisely, its edges $e_i \subset E \cap H^{\Delta }_i$ move in a self-parallel manner and at individual speeds $w_i = \frac{1}{\sin \alpha _i} > 0$, where $\alpha _i$ is the dihedral angle formed by *E* and the facet(s) of ${\mathcal{Q}}$ corresponding to the offset plane $H^{\Delta }_i$. This planar offsetting process traces out the so-called weighted straight skeleton [7, 13, 21] of ${\mathcal{P}}$ with respect to the edge weights $w_i$. Note that the particular position of the sectional plane *E* influences both ${\mathcal{P}}$ and its weights $w_i, \ldots , w_m$, in a way such that the resulting skeleton, $\text{ SK }(v)$, remains combinatorially unaffected: A polygon vertex *u* shared by edges $e_i$ and $e_j$ moves in an angle bisector plane, $B_{ij}$, of $H^{\Delta }_i$ and $H^{\Delta }_j$ (which is independent of $\Delta$ and *E*). Therefore, *u* traces out a skeleton arc along the line $B_{ij} \cap E$. In fact, the skeletons obtained for different choices of *E* are radial projections of each other (with respect to the polytope vertex *v*, where all such bisector planes $B_{ij}$ pass through).

Barequet et al. [12] proposed the interesting idea of using the incidence relations of the weighted straight skeleton $\text{ SK }(v)$ to resolve the vertex *v*. We will elaborate on the properties of this skeleton in some more detail here.

Weighted straight skeletons are not unique when degenerate conditions arise. Edge events that involve parallel polygon edges can cause ambiguity; see e.g. [13]. (For unweighted polygons, both edge events and split events always yield a unique offset boundary, at least in the classical setting.^6^) However, edge weights are not independent in our case, which leads to the special property below that we will prove first. Let us call a polygon vertex *convex*, *reflex*, or *flat*, respectively, if its incident interior angle is smaller, larger, or equal to $\pi$. We observe that a vertex of our sectional polygon ${\mathcal{P}}$ is convex (respectively, reflex) if and only if its defining edge in the polytope ${\mathcal{Q}}$ is convex (respectively, reflex). In other words, the convexity status of a vertex of ${\mathcal{P}}$, or of any of its offset polygons, is not influenced by the particular position of the sectional plane *E*.

#### Lemma 4.1

In the edge events that occur in the construction of $\text{ SK }(v)$, only *convex* new vertices are created in the offset polygon(s).

#### Proof

**Fig. 5:**
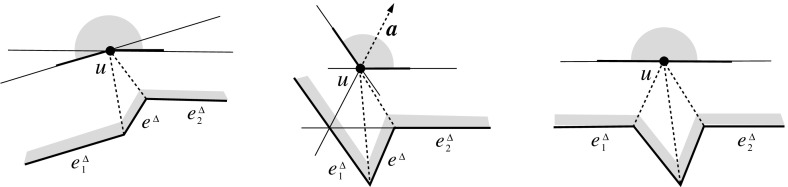
The vertex *u* is not generated in the *left* and the *right* case, respectively. In the case shown in the *middle*, the skeleton faces sharing the arc *a* continue in a monotone way

Suppose that a nonconvex vertex, *u*, is created in an edge event; see Fig. 5(left). Then some polygon edge *e* has to shrink to length zero, and the offsets $e^{\Delta }_1$ and $e^{\Delta }_2$ of two other edges $e_1$ and $e_2$ get in touch at *u*, forming an interior angle of at least $\pi$. Let us assume first that, before the event, $e^{\Delta }_1$ and the offset $e^{\Delta }$ of *e* define a *convex* interior angle (as drawn in the figure). We fix the sectional plane *E* orthogonal the line segment $\overline{uv}$, and such that $\overline{uv}$ is of unit length. Then the supporting line $\ell _i$ of edge $e_i$ is at distance $\cot \alpha _i$ from *u*. Note that we have $\alpha _1, \alpha _2 < \frac{\pi }{2}$. As $e_i$ shifts with speed $\frac{1}{\sin \alpha _i}$, the line $\ell _i$ reaches *u* at time $\cos \alpha _i$. But we have $\alpha _1 \ne \alpha _2$ in our case, by the existence of $e^{\Delta }$ before the event. We conclude that $\ell _1$ and $\ell _2$ cannot reach *u* at the same time, which is necessary for the occurrence of *u* as an offset vertex. A similar argument for the nonexistence of *u* applies when the offsets $e^{\Delta }_1$ and $e^{\Delta }$ form a *reflex* interior angle (this case is not shown in the figure). It remains to recall that the vertex structure of $\text{ SK }(v)$ is the same for all choices of the plane *E*. $\square$

Note that when a (convex) vertex *u* is created in an edge event, then $\alpha _1 = \alpha _2$ holds for the position of *E* chosen above; see Fig. 5(middle). To be precise, in the degenerate case where two or more edges vanish at the same time in the event, rather than a single edge *e*, the vertex *u* may even be flat in order to have $\alpha _1 = \alpha _2$; see Fig. 5(right). But then we must have $\ell _1 = \ell _2$, instead of mere parallelism of these supporting lines, such that $e^{\Delta }_1$ and $e^{\Delta }_2$ merge into a single edge, and *u* actually is not created as a polygon vertex. (Observe that *u* leaves no further trace, but still represents a node of $\text{ SK }(v)$.) As a consequence, Lemma 4.1 still holds. In particular, the afore-mentioned ambiguous case for parallel edges is excluded (where we have $\ell _1 \ne \ell _2$, hence $\alpha _1 \ne \alpha _2$).

In summary, there is a unique way to proceed in the construction of $\text{ SK }(v)$ after each event. That is, we have a similar behavior as in the unweighted case [4].

The inner arcs of $\text{ SK }(v)$ form a tree such that exactly one face $g_i$ for each edge $e_i$ of ${\mathcal{P}}$ is present, unless the degenerate case above occurs and certain vertices in the offset polygon ‘flatten out’. $\text{ SK }(v)$ then is a forest where some collinear edges of ${\mathcal{P}}$ border the same skeleton face. Interestingly, the faces of $\text{ SK }(v)$ have the following connectivity behavior, which we explain below: They are *monotone polygons*, in particular, the intersection of a face $g_i$ with any line normal to the defining edge $e_i$ is connected. This property is well known for the unweighted straight skeleton [4], but does not hold for weighted straight skeletons in general, unless the underlying polygon is convex; see e.g. [7, 13].

#### Lemma 4.2

The weighted straight skeleton $\text{ SK }(v)$ is a unique structure (up to radial projection from *v*). Moreover, its faces are monotone in the direction of their defining polygon edges.

To see the monotonicity property, we observe that non-monotonicity always arises in the neighborhood of skeleton nodes created by so-called *sticking events*; see e.g. [31]. These are edge events where an arc incident to a reflex polygon vertex is involved. However, when a node *u* of $\text{ SK }(v)$ is generated in a sticking event, then we must have the situation in Fig. 5(middle), by Lemma 4.1. The skeleton construction then continues with an arc *a* starting from *u*, in a way such that the two faces which share *a* are monotone in the required directions.

By Lemma 4.2, the improvements in [15, 16] of the subquadratic-time algorithm in [21] for constructing straight skeletons are applicable to $\text{ SK }(v)$; they are based on monotone faces. As a consequence, $\text{ SK }(v)$ can be computed in (roughly) $O(m^{\frac{4}{3}})$ time, when *m* is the degree of *v*. The next assertion, which has been stated informally in [12], shows the relevance of $\text{ SK }(v)$ for resolving the pointed vertex *v*.

#### Lemma 4.3

There is a valid offset surface for *v* that radially projects to $\text{ SK }(v)$.

#### Proof

Clearly, the domain for such a surface $\Sigma$ has to be the spherical polygon ${\mathcal{S}}$ obtained by radially projecting the polygon ${\mathcal{P}}$ onto a sphere *U* centered at *v*. (${\mathcal{S}}$ is contained in an open hemisphere of *U* now, namely, in the radial projection of the sectional plane *E*.) Consider an arbitrary point $x \in {\mathcal{S}}$, and denote with $x'$ its radial projection to ${\mathcal{P}}$. We define the surface $\Sigma$ by putting $\Sigma (x) = H^{\Delta }_i(x)$ if and only if $x'$ lies in the unique (closed) face $g_i$ of $\text{ SK }(v)$ that is bordered by the edge $e_i$ of ${\mathcal{P}}$. It remains to prove that $\Sigma$ satisfies the conditions in Definition 3.1. Obviously, $\Sigma$ is radially visible from *v* and fulfills (1) and (2) by construction. To see that $\Sigma$ is also continuous, (3), consider any inner arc *a* of $\text{ SK }(v)$, and let $g_i$ and $g_j$ be the two skeleton faces incident to *a*. Arc *a* is contained in an angle bisector plane $B_{ij}$ of the offset planes $H^{\Delta }_i$ and $H^{\Delta }_j$, and $B_{ij}$ passes through the vertex *v*. This implies that arc *a* radially projects to the line $H^{\Delta }_i \cap H^{\Delta }_j$. (Note that these two planes cannot be identical, by the existence of *a*.) We conclude that for each $x \in {\mathcal{S}}$ with $x' \in a$, we must have $H^{\Delta }_i(x) = H^{\Delta }_j(x)$, that is, the facets of $\Sigma$ fit continuously. $\square$

Lemma 4.3 implies Corollary 3.4 in Sect. 3.2, for the special case of pointed polytope vertices *v*. In particular, each polytope facet $f_i$ incident to *v* gives rise to a single and unbounded facet $f^{\Delta }_i$ in the surface $\Sigma$ above. In the degree-7 vertex example discussed in Sect. 3.2, the orphan-free surface in Fig. 3(right) is the one of all solutions that corresponds to $\text{ SK }(v)$.

**Fig. 6:**
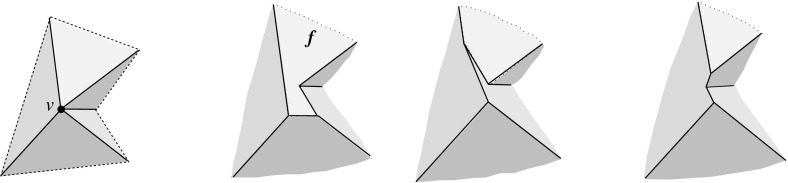
The vertex *v* splits in a unique way

We remark that Lemma 4.3—in conjunction with Lemma 4.2—puts some restriction on valid offset surfaces. This is demonstrated in Fig. 6. If the degree-5 vertex *v* (left) could be resolved in two different ways, then the two surfaces (middle, left/right) were obtained. The former surface cannot come from $\text{ SK }(v)$, because the face *f* is not monotone there. So it must be the latter one, which (unlike the former surface) does not result from a self-parallel polygon offsetting process, not even for arbitrary edge weights. We conclude that there is a unique solution, which is the one shown in Fig. 6(right).

There is another and more well-known projection surface for weighted straight skeletons of a polygon ${\mathcal{P}}$, called the *skeleton roof*; see e.g. [7, 21]. This surface does not stem from any offset planes, but rather from the planes $L_i$ that result from lifting each point $x \in {\mathcal{P}}$ vertically, by $\frac{1}{w_i}$ times the (signed) distance of *x* to the supporting line $\ell _i$ of an edge $e_i$ of ${\mathcal{P}}$.

**Fig. 7:**
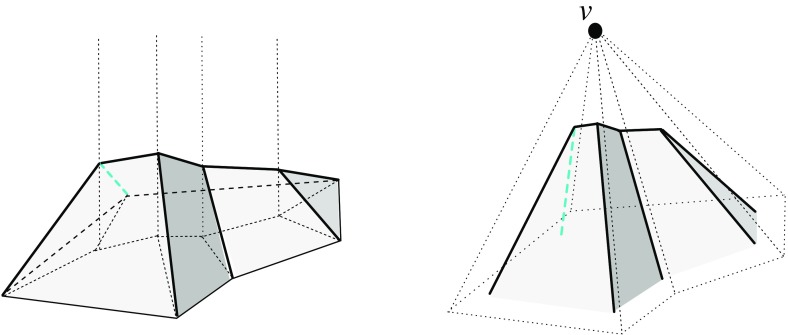
The skeleton roof (*left*) and its corresponding offset surface (*right*)

The skeleton roof corresponding to $\text{ SK }(v)$, and the offset surface $\Sigma$ for *v* in Lemma 4.3, have the same convex/reflex structure of their edges: The convexity status of a surface edge is uniquely determined by the interior angle formed by the respective two edges of ${\mathcal{P}}$. Figure 7 offers an illustration. Note that Lemma 4.1 implies that all *valleys* of this roof (i.e., reflex edges) have to start at the boundary of ${\mathcal{P}}$, a property that in the unweighted case is characterizing for the skeleton roof, among all possible roofs for ${\mathcal{P}}$; see [4]. As a consequence, a similar restriction for reflex edges can be used to distinguish the offset surface $\Sigma$ from all other possible solutions. We will see in Sect. 7 that a generalization of this property to one dimension higher (Lemma 7.4) leads to an alternative characterization of 3D straight skeletons, equivalent to their procedural definition in Sect. 5.

We observe finally that when the edge weights $\cot \alpha _i$ (instead of $\frac{1}{\sin \alpha _i}$) are used for the sectional polygon ${\mathcal{P}}$, then the resulting skeleton roof degenerates to a pyramid with apex *v* and base ${\mathcal{P}}$; compare the proof of Lemma 4.1. This is the object we obtain when we cut off the vertex *v* from ${\mathcal{Q}}$ with the plane *E*, because the planes $L_i$ defining the roof are now the facet planes of *v*.

### Bisector Graphs

In this subsection, we discuss a two-dimensional reduction of the vertex resolution problem for arbitrary polytope vertices.

Non-pointed polytope vertices are more complicated to deal with. For example, if a vertex *v* of the polytope ${\mathcal{Q}}$ is a saddle vertex, then a single sectional plane (like in the preceding subsection) does not suffice to intersect all the edges incident to *v*. Using two sectional planes leads to one or more unbounded polygons of intersection with ${\mathcal{Q}}$ in either plane. If the degree of *v* is high, these polygons can be arbitrarily complex, having a large number of convex and reflex vertices. Though the weighted straight skeleton inside each such polygon can be defined and computed much like in the (bounded) case before, we now face the task of combining several skeletons that stem from two different planes. This inherits the problem of merging straight skeletons, which is unsolved so far. In fact, a solution would imply a novel divide & conquer method for computing straight skeletons.

This situation can be circumvented when a sectional sphere *U* as in Sect. 3 is used for a two-dimensional reduction. In the sequel, let *v* be an arbitrary vertex of the polytope ${\mathcal{Q}}$. We first consider the spherical polygon ${\mathcal{S}}= U \cap {\mathcal{Q}}$, which may have a more general shape now: The boundary of ${\mathcal{S}}$ stays connected as long as *v* is non-touching (for instance, a saddle vertex), but it necessarily disconnects if *v* is a touching vertex, and ${\mathcal{S}}$ needs not be simply connected any more, and even can disconnect itself.

Still, a valid offset surface for *v* always exists. In fact, Definition 3.1 and Theorem 3.3 from Sect. 3 generalize directly to more general domains ${\mathcal{S}}$. The only difference introduced by a touching vertex *v* concerns the facet planes for *v*. In addition to the facets of ${\mathcal{Q}}$ that have *v* as a vertex, there exist facets $f_i$ of ${\mathcal{Q}}$ now which are only touched by *v*, and whose offset planes $H^{\Delta }_i$ have to be taken into account for the arrangement ${\mathcal{A}}(v)$ as well. Note that *v* may touch a facet $f_i$ singularly, or an edge or a facet where *v* is a vertex may touch $f_i$. In any case, the offset plane arrangement ${\mathcal{A}}(v)$ is well defined, such that all the results from Sect. 3 can be extended.

Let now $\Sigma$ be a valid offset surface for *v*. We observe that $\Sigma$ can contain holes and can be disconnected, if the same happens for the spherical polygon ${\mathcal{S}}$. Our interest is in the incidence structure of the edges of $\Sigma$, which is encoded in the set of non-differentiability of the radial function over ${\mathcal{S}}$ whose image is $\Sigma$. This set defines a geometric graph in the interior of ${\mathcal{S}}$, which we term the *bisector graph*, $G_v(\Sigma )$, for *v* and $\Sigma$. Trivially, the arcs of $G_v(\Sigma )$ pairwise do not cross, but rather partition ${\mathcal{S}}$ into maximal subdomains (called *regions*) where the function $\Sigma (x)$ is differentiable. In geometric terms, the regions of $G_v(\Sigma )$ are the radial projections of the facets of $\Sigma$.

The arcs of $G_v(\Sigma )$ are subsets of great circles $b_{ij}$ of the form $b_{ij} = B_{ij} \cap U$, where $B_{ij}$ is the angle bisector plane through *v* of the offset planes $H^{\Delta }_i$ and $H^{\Delta }_j$. Notice that the arcs of ${\mathcal{S}}$ are *not* considered to be part of $G_v(\Sigma )$. The nodes of ${\mathcal{S}}$ are therefore of degree 1 in $G_v(\Sigma )$, and will be called its *leaves*. The inner nodes of $G_v(\Sigma )$ are of degree 3 or higher, where a higher degree can occur for two reasons: the presence of orphan facets in $\Sigma$ as in Fig. 4(left), or because the offset arrangement ${\mathcal{A}}(v)$ is not generic.

**Fig. 8:**
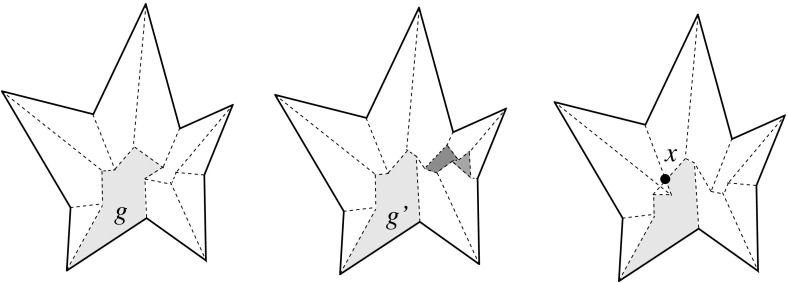
Bisector graphs with a tree structure (*left*), or containing cycles which correspond to orphan facets (*middle*, *dark gray*). Embedding a different tree leads to crossing arcs (*right*)

Figure 8(left/middle) displays the bisector graphs for two valid offset surfaces of a vertex *v* with degree 10. The domain ${\mathcal{S}}$ is a pentagonal star, sufficiently small to be almost flat. Examples like this can be duplicated and combined, to show that the number of possible valid offset surfaces is as large as $2^{\Omega (m)}$ in the worst case, when *v* is of sufficiently high degree *m*. This is already true for surfaces having the forest structure as in Corollary 3.4, that is, for bisector graphs without orphan regions: In Fig. 8, the regions *g* and $g'$ are already different. In such an example of exponential behavior, *v* can either be a touching vertex, being the common apex of $\Theta (m)$ pyramids with pentagonal stars as bases, or a pointed vertex when the stars are joined into a connected spherical polygon. If orphan regions are allowed, then there exist examples (based on Fig. 27) where *v* splits into $\Theta (m^2)$ surface vertices. The graph in Fig. 8 (right) contains crossing arcs, and therefore is not a bisector graph. Various (spherical) illustrations of bisector graphs are given in Sect. 6, along with the events they represent in the construction of 3D straight skeletons.

Our next aim is an extension of Corollary 3.4 that includes the touching vertex type. To this end, we call a bisector graph $G_v(\Sigma )$ *outerplanar* (with respect to the spherical polygon ${\mathcal{S}}$ for *v*) if all regions of $G_v(\Sigma )$ are adjacent to the boundary of ${\mathcal{S}}$. Observe that $G_v(\Sigma )$ is outerplanar if and only if $\Sigma$ contains no orphan facets. The graph then captures the features of an offset surface which are desirable for the reasons mentioned in Sect. 3.2. Revisiting the proof of Corollary 3.4, we see that the surface $\Sigma$ considered there already yields a bisector graph $G_v(\Sigma )$ with the required properties. Denote with $c \ge 0$ the number of elementary cycles in $G_v(\Sigma )$.

#### Corollary 4.4

Let *v* be an arbitrary vertex of the polytope ${\mathcal{Q}}$, and let *m* be the number of facets that contain *v*. There exist (orphan-free) valid offset surfaces $\Sigma$ for *v* such that $G_v(\Sigma )$ is an outerplanar graph with at most $m+2(c-1)$ inner nodes. In the generic case, all these nodes have degree 3.

Cycles in outerplanar bisector graphs stem from the shape of the domain ${\mathcal{S}}$ and from the sphere topology. Such graphs may also be disconnected, even within a single connected component of ${\mathcal{S}}$. In the illustrations given in Sect. 6, $G_v(\Sigma )$ is always outerplanar.

To obtain a canonical bisector graph for a polytope vertex *v*, the *minimum surface* $\Sigma ^-$ or the *maximum surface* $\Sigma ^+$ (defined at the end of Sect. 3.2) can be utilized, or their orphan-free variants when outerplanarity is required. Another unique bisector graph for *v*, which is always outerplanar, is the so-called *spherical skeleton*, $G^*_v$, of ${\mathcal{S}}$ on the sectional sphere *U*, introduced in [9]. This skeleton is defined by a carefully tuned shrinking process for ${\mathcal{S}}$, based on the moving offset planes $H^{\Delta }_i$. The task of constructing $G^*_v$ is somewhat involved, concerning the arising events which are more numerous than in the case of planar straight skeletons. We decided not to include the details here, and refer the interested reader to [9] for a description of this material instead. The spherical skeleton $G^*_v$ can be computed in $O(m^2 \log m)$ time, where *m* is the number of facets of ${\mathcal{Q}}$ that contain *v*.

Observe that the existence of $G^*_v$ implies a general proof of existence for valid offset surfaces. In fact, $G^*_v$ is a generalization to the sphere of the weighted straight skeleton $\text{ SK }(v)$ in Sect. 4.1. This shows $G^*_v \ne G_v(\Sigma ^+)$ in general, by Fig. 3(right) that corresponds to $G^*_v$, and Fig. 4(right) that corresponds to $G_v(\Sigma ^+)$. There also exist examples for $G^*_v \ne G_v(\Sigma ^-)$. In contrast to $\text{ SK }(v)$, the use of a sectional sphere, rather than a sectional plane, obviates the need for weighting the arcs of the obtained spherical polygon.

To select more than one bisector graph for *v*, the offset arrangement ${\mathcal{A}}(v)$ can be used directly. ${\mathcal{A}}(v)$ contains $\Theta (m^3)$ cells, which can be computed in optimal $\Theta (m^3)$ time [20]. This is also the worst-case time complexity for extracting the first outerplanar bisector graph with the cell adding technique in Theorem 3.3: It is not hard to find an example where a cubic number of cells have to be added. On the other hand, ${\mathcal{A}}(v)$ implicitly encodes all possible solutions (which can be exponentially many even when only outerplanar graphs are sought), thus providing all possible choices of how to proceed in the offsetting process.

A computationally simpler and still flexible approach, and the one we have implemented for the 3D straight skeleton construction, is the following. By Corollary 4.4, we can enumerate all combinatorially different outerplanar graphs which are relevant, and check whether they are bisector graphs for the polytope vertex *v* under consideration. Being easy to implement, this method is plausible when the degree *m* is a constant, independent of the number *n* of facets of the input polytope. Indeed, most solids can be approximated accurately by (boundary-meshed) polytopes with vertices of small constant degree. Also, in the generic case, the shrinking process can only create offset polytope vertices of degree $\le$8, as it will turn out in Sect. 6.

The graph enumeration is facilitated by the following observations. Let ${\mathcal{S}}_1, \ldots , {\mathcal{S}}_t$ be the connected components of the spherical polygon ${\mathcal{S}}$ for the vertex *v* to be resolved. We can treat each component ${\mathcal{S}}_k$ separately, and moreover, adapt to the properties of ${\mathcal{S}}_k$. If the boundary of ${\mathcal{S}}_k$ is connected then Corollary 3.4 applies, and attention can be restricted to graphs that are forests. The same can be done if *v* is a non-touching vertex, where we also have only one connected component. Let now $m_k$ be the number of arcs of ${\mathcal{S}}_k$, and consider the system $(b_{ij})$, ${1 \le i < j \le m_k}$, of great circles, obtained by intersecting the sphere *U* with the angle bisector planes $B_{ij}$ associated with ${\mathcal{S}}_k$. The next lemma provides a criterion for recognizing whether a given candidate graph is a bisector graph.

#### Lemma 4.5

Let *G* be an outerplanar graph for ${\mathcal{S}}_k$. Then *G* is a bisector graph for ${\mathcal{S}}_k$ and *v* if and only if (1) all inner nodes of *G* have degree $\ge$ 3, and (2) the arcs and nodes of *G* can be embedded on the respective components of the circle arrangement $(b_{ij})$ inside ${\mathcal{S}}_k$ without self-crossings.

#### Proof

If *G* fulfills conditions (1) and (2) then a valid offset surface with facets from the offset planes $H^{\Delta }_1, \ldots , H^{\Delta }_{m_k}$ can be constructed, similar as in the proof of Lemma 4.3. Conversely, any bisector graph has to fulfill (1), because the vertices of a surface are of degree at least 3. Assume now that *G* does not embed inside ${\mathcal{S}}_k$ without arc crossings, and refer to Fig. 8(right). We claim that the offset planes above now give a surface for *G* that is not radially visible from *v*, implying that *G* cannot be a bisector graph: Let two arcs in the embedding of *G* cross at the point $x \in {\mathcal{S}}_k$. Then the ray from *v* and through a suitable point in the neighborhood of *x* intersects two surface facets (rather than only one) in their interiors. $\square$

Condition (2) in Lemma 4.5 can be tested in $O(m_k \log m_k)$ time, for example, by using a generalized plane sweep algorithm for line segment intersection; see e.g. [17]. As a useful byproduct, the geometric embedding of *G* provides us with extra information, such that only *connected* graphs with inner nodes of degree *exactly 3* need to be generated: Arcs of *G* missing in the corresponding bisector graph, and thus causing its disconnectedness, reveal themselves by the identity of the respective offset planes. (Sometimes two arcs of *G* which are incident to such a ‘flat’ arc have to be concatenated into a single arc of the bisector graph; this is reflected by their containment in the same great circle $b_{ij}$.) Moreover, nodes of degree $\ge$4 in the bisector graph are witnessed by arcs of *G* of length zero. These observations make the enumeration particularly easy when *G* is a forest, where it suffices to generate all labeled and unrooted binary trees with $m_k$ leaves [26].

In summary, a universal engine for vertex resolution is obtained, which works for arbitrary polytope vertices and in all degenerate cases. This ‘vertex splitter’ is useful not only for initially splitting the higher-degree vertices of the input polytope ${\mathcal{Q}}$, but also for handling all the events that arise later during the offsetting process for ${\mathcal{Q}}$. Even any *multiple event* can be processed, i.e., a combination of events of possibly different types, which take place at the same point in space, and which can be arbitrarily complex. (For example, when we shrink the complement of the polytope shown in Sect. 2, the vertex *v* gives rise to a multiple event. See also Fig. 27 in Sect. 8.) In fact, the type of an event needs *not* be known in advance in order to process it correctly. Still, we will give a complete categorization of (non-initial) events in Sect. 6, under the assumption that the offset planes behave generically.

## Straight Skeletons in Three-Space

After having settled some basic questions about mitered offset surfaces for general nonconvex polytopes, we can now return to the main concern of this paper: the construction of 3D straight skeletons for such input polytopes. We will maintain full generality in this section, to point out the general validity of the results. In Sect. 6, we will have to introduce a generic condition, to be able to categorize the skeleton construction events in a transparent way.

### Construction Process

Like in two-dimensions, the skeleton construction complies with the shrinking process for the given polytope ${\mathcal{Q}}$, and is driven by so-called *events*. These are combinatorial changes in the boundary structure of the offset polytope ${\mathcal{Q}}^{\Delta }$. Events take place only if there is a change in the number of offset planes $H^{\Delta }_i$ that intersect in the same vertex of ${\mathcal{Q}}^{\Delta }$. This number is at least three; the results in Sect. 3 ensure that only valid polytopes as defined in Sect. 2 are generated. When an event happens at vertex *v*, then four or more offset planes participate, which when shifted further, constitute the respective offset arrangement ${\mathcal{A}}(v)$.

Initially, for infinitesimally small $\Delta$, various events will have happened simultaneously in general, which split higher-degree vertices of ${\mathcal{Q}}$ into vertices of smaller degree. (We will call such events the *initial events*, to distinguish them from the *non-initial events* that occur later in the offsetting process for ${\mathcal{Q}}$.) Then, when $\Delta$ increases, between any two consecutive events the boundary of ${\mathcal{Q}}^{\Delta }$ keeps its incidence structure while offsetting. Each facet, edge, and vertex of ${\mathcal{Q}}^{\Delta }$ traces out a certain part of a *cell*, or *sheet*, or *spoke*, respectively, as we shall name these skeleton components. The edges of ${\mathcal{Q}}^{\Delta }$ move in angle bisector planes, and the vertices of ${\mathcal{Q}}^{\Delta }$ move along trisector lines, which are the common intersections of three bisector planes. This implies that a piecewise-linear structure is being constructed.

Every event is associated with a vertex *v* of ${\mathcal{Q}}^{\Delta }$ where the number of offset planes that contain *v* undergoes a change. The vertex *v* becomes part of the skeleton in the event, being an endpoint of certain skeleton spokes. We will call such endpoints the *corners* of the skeleton. Note that events may happen *simultaneously* also for a fixed value $\Delta > 0$, such that $k \ge 2$ different vertices $v_1, \ldots , v_k$ of ${\mathcal{Q}}^{\Delta }$ are involved at the same time in different events. Now, the way how each such vertex $v_i$ gets resolved fixes the combinatorics and the geometry of the boundary of ${\mathcal{Q}}^{\Delta }$, for infinitesimally increased $\Delta$. This, in turn, uniquely determines how the skeleton construction for the polytope ${\mathcal{Q}}$ will proceed for larger $\Delta$, till the next event is encountered.

By the results in Sects. 3 and 4, the resolution of a vertex $v_i$ is always possible via its offset arrangement ${\mathcal{A}}(v_i)$, but this may be an ambiguous process. This concerns the initial events, but also the non-initial ones, especially when a non-generic or multiple event occurs. (Recall that a multiple event refers to a single vertex, unlike simultaneous events.) Still, Definition 3.1 guarantees that any possible (infinite) sequence of offset polytopes ${\mathcal{Q}}^{\Delta }$, for $\Delta$ growing from 0 to $\infty$, is totally ordered by inclusion. Moreover, the boundary of ${\mathcal{Q}}^{\Delta }$ changes continuously with $\Delta$, such that each point $x \in {\mathcal{Q}}$ is swept over by the shrinking polytope boundary exactly once. This implies that the offsetting process cannot ‘cycle’ or lead to overlapping skeleton parts, and that ${\mathcal{Q}}^{\Delta }$ (which before might have disconnected into other components having vanished already) eventually has to collapse to volume zero, in a final event. In summary, a piecewise-linear cell complex inside ${\mathcal{Q}}$ is constructed; see Figs. 10 and 29 for illustrations. We are now ready to state a main theorem of this paper.

#### Theorem 5.1

Let ${\mathcal{Q}}$ be a polytope in ${\mathbbm {R}}^3$ as defined in Sect. 2. Any of the (at least one) mitered offsetting processes for ${\mathcal{Q}}$ as in Definition 3.1 terminates with the construction of a piecewise-linear decomposition of ${\mathcal{Q}}$.

### Facial Structure, Topology, and Size

In the decomposition a straight skeleton defines for a polytope ${\mathcal{Q}}$, the cells are *nonconvex* sets in general. Studying their structure is therefore a nontrivial task.

We start by arguing that skeleton cells are always bordered by some polytope facet. The *skeleton cell* of a facet $f_i$ of ${\mathcal{Q}}$ is defined as the total volume swept over by the boundary part of ${\mathcal{Q}}^{\Delta }$ that comes from the offset plane $H^{\Delta }_i$ for $f_i$. This volume is a *connected* set, and the reason is that $H^{\Delta }_i$ contributes to the boundary of ${\mathcal{Q}}^{\Delta }$ in a continuous way: By definition of the vertex resolution process, $H^{\Delta + \varepsilon }_i$ can yield a facet in ${\mathcal{Q}}^{\Delta + \varepsilon }$ only if $H^{\Delta - \varepsilon }_i$ did so in ${\mathcal{Q}}^{\Delta - \varepsilon }$, for infinitesimal $\varepsilon$ with $\Delta> \varepsilon > 0$. In particular, once having stopped contributing to the polytope boundary, an offset plane cannot reappear, because it does not participate in any future vertex resolutions.

Observe that different (but then coplanar) facets of ${\mathcal{Q}}$ can border the same cell, if they define the same offset plane. Similarly, a single offset facet can lose simple connectedness or split into orphan pieces, as in Figs. 4 and 8. The produced skeleton cell, *C*, then can split into several interior-connected *orphan cells*, though *C* still stays connected through the vertices that have been resolved. (Fig. 27 offers an illustration for the planar case.) Such probably undesirable artifacts can be avoided, when abiding by the orphan-free offset surfaces in Corollary 4.4. Unavoidable is the occurrence of *tunnels* in skeleton cells and of *holes* in skeleton sheets, e.g., when ${\mathcal{Q}}$ has facets with holes. Simple examples exist in this case; see Fig. 29.

In any case, the cells share another property which is helpful when using 3D straight skeletons as a partitioning structure: Each cell *C* is *monotone*, in the sense that the intersection of *C* with any line normal to its defining facet(s) of ${\mathcal{Q}}$ is connected or empty. This follows from Lemma 7.2 in Sect. 7, which covers a more general class of cell complexes for ${\mathcal{Q}}$ introduced there. The monotonicity implies the absence of *voids* in skeleton cells, even when the polytope ${\mathcal{Q}}$ itself is not void-free.

We now take a closer look at the *facial structure* present in a straight skeleton for ${\mathcal{Q}}$. The main fact determining the incidence structure is that, in the offset polytope ${\mathcal{Q}}^{\Delta }$, all vertices are of degree at least 3 for any value of $\Delta$. This implies that each spoke has 3 incident sheets and cells, respectively, or more in non-generic cases (which we do not discuss in detail here), but not possibly only 2.

**Fig. 9:**
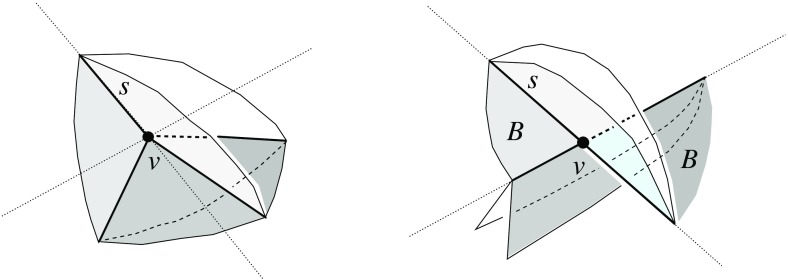
The two possible (generic) incidence structures at a straight skeleton corner *v*

Consult Fig. 9. Let $\ell$ be the trisector line that supports a spoke *s*, and consider an endpoint *v* of *s* which is an *inner corner* of the skeleton (that is, *v* is not a vertex of ${\mathcal{Q}}$). Another trisector line $\ell ' \ne \ell$ has to pass through *v*. The six involved bisector planes, one triple for $\ell$ and one for $\ell '$, now can be pairwise different or not. In the former case (left), *v* is incident to 6 sheets, 4 spokes that span 3-space, and 4 cells, like in a convex cell complex.

In the latter case (right), one plane in the triple for $\ell$ identifies with a plane in the triple for $\ell '$. (No other pair can identify, by $\ell ' \ne \ell$). This plane, call it *B*, contains the lines $\ell$ and $\ell '$, each of which defines two collinear spokes of *v*. These 4 spokes can span only 2 diametral sheets contained in *B*, but they span 4 other sheets that stem from the two bisector planes different from *B* in each triple. The latter sheets therefore have *v* as a ‘flat’ corner. Again, 4 cells meet at *v*, but there are two cells, say $C_1$ and $C_2$, each of which is supported by *both* sides of the plane *B*, having a double-adjacency there. Note that the resulting cell complex is still *face-to-face*, because *v* is a corner in all participating faces. Therefore, this geometric anomaly implies no inconsistency with the local incidence structure given in a convex face-to-face cell complex. The events shown in Figs. 12, 13, and 21 lead to such an interesting constellation, in a generic way. In conclusion, from an implementation point of view, any of the various available data structures for storing convex cell complexes can be used for 3D straight skeletons.

Geometrically, when a skeleton cell is considered as a separate polytope, it can have several kinds of vertices, including the reflex and the saddle type. Touching vertices occur as well, namely, as corners that concatenate orphan parts of the same skeleton cell. However, in the geometric degeneracy described above, the vertex *v* is not a touching vertex for the cells $C_1$ and $C_2$.

Returning to topological issues, let us study the *spoke graph* of a 3D skeleton next, i.e., the graph formed by its spokes and corners. Whereas skeleton cells are always connected, this is not true for the spoke graph. Two examples can be found in Sect. 6.1. They reflect that vertices of ${\mathcal{Q}}^{\Delta }$ which disappear in the interior of a polytope facet or edge (in the events ${\mathcal{E}}_6$ and ${\mathcal{E}}_5$) are responsible, as they leave no further trace. Such situations arise naturally, in contrast to the 2D case: The planar straight skeleton cannot disconnect, unless geometric degeneracies are present in the input polygon; see Fig. 5(right) and Sect. 8.1. As a comforting fact, we have:

#### Lemma 5.2

Consider any straight skeleton of the polytope ${\mathcal{Q}}$. In the corresponding spoke graph, each component is connected to ${\mathcal{Q}}$’s boundary.

#### Proof

Suppose the contrary, and let *I* be some isolated component of the spoke graph that is not connected to the boundary of ${\mathcal{Q}}$. Then there exists some closed surface which separates *I* from ${\mathcal{Q}}$’s boundary, but does not intersect any spoke or sheet in the straight skeleton for ${\mathcal{Q}}$. Hence there must be a cell with a void which entirely surrounds *I*, and the cells therein (whose existence is witnessed by the spokes in *I*) are not bordered by the boundary of ${\mathcal{Q}}$. These are contradictions to two of the properties of a skeleton cell, mentioned at the beginning of this subsection. $\square$

By Lemma 5.2, graph connectedness is regained when the spoke graph is joined with the graph formed by the edges of ${\mathcal{Q}}$, provided the latter graph is connected. (This can always be achieved when ${\mathcal{Q}}$ has no voids, by triangulating the boundary of the input polytope ${\mathcal{Q}}$ after the skeleton construction). We point out that this connectivity property is not shared by the trisector arcs of the *medial axis* of ${\mathcal{Q}}$, which has a more complex topology; see e.g. [29]. This complicates the application of tracing algorithms for computing 3D medial axes. We will address such algorithms in Sect. 7.

Naturally, the *combinatorial complexity* of a 3D skeleton is an interesting quantity. It is defined as the total number of cells, sheets, spokes, and corners of the skeleton. Let the polytope ${\mathcal{Q}}$ have *n* facets. The number of cells is at most *n*, as each facet borders a unique connected cell. However, a cell can consist of various orphan cells connected via corners. The number of corners is trivially bounded from above by ${n \atopwithdelims ()4}$, because 4 offset planes have to concur in the event that constructs a particular corner, and this can happen only once for each quadruple of planes. This implies a bound of $O(n^4)$ on the size of any straight skeleton; the numbers of corners, spokes, and sheets, respectively, are linearly related. (See also the result in Sect. 7.3.)

**Fig. 10:**
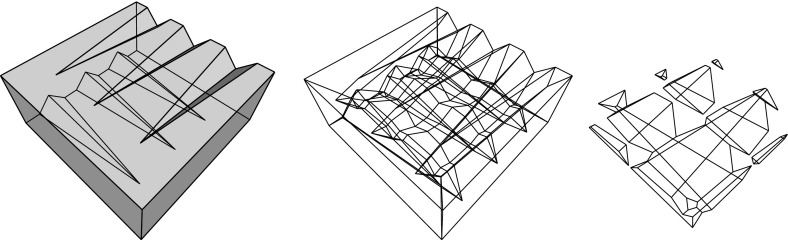
The polytope ‘Pizza Box’ has a cross pattern of $\Theta (n)$ long notches (*left*). The cell of the top facet is adjacent to the cell of the bottom facet in $\Theta (n^2)$ skeleton sheets (*middle*). The offset polytope splits into a quadratic number of pieces, thus having $\Theta (n^2)$ facets (*right*)

Let now $B_{ij}$ be the bisector plane of the offset planes $H^{\Delta }_i$ and $H^{\Delta }_j$. A single trisector line $\ell _{ijk} = B_{ij} \cap B_{jk}$ can contribute at most $n-2$ spokes to the skeleton, because $\ell _{ijk}$ contains at most one corner for each index different from *i*, *j*, *k*. By a similar index count, a single bisector plane $B_{ij}$ contributes at most $\frac{1}{2} (n^2-3n+4)$ sheets—the maximum number of planar faces in an arrangement of $n-2$ lines. These bounds are tight in order, as the example in Fig. 10 shows. This implies that a single cell can have a combinatorial complexity of $\Omega (n^2)$, even without decomposing into orphan cells. Figure 10 also reflects that the number of facets of the offset polytope ${\mathcal{Q}}^{\Delta }$ can increase quadratically. However, the overall size of a straight skeleton has a cubic upper bound, at least in the customary and probably most useful setting, by the following theorem which we will prove in Sect. 7.2.

#### Theorem 5.3

Let ${\mathcal{Q}}$ be a boundary-connected polytope in ${\mathbbm {R}}^3$ having *n* facets. The combinatorial complexity of any straight skeleton for ${\mathcal{Q}}$ is $O(n^3)$, provided it has been constructed with orphan-free vertex resolution.

Clearly, the result also holds when ${\mathcal{Q}}$ consists of several connected components. Notice that ${\mathcal{Q}}$ may contain tunnels, but voids are disallowed. No assumptions of non-degeneracy are required. Previously only the trivial upper bound $O(n^4)$ has been known. We stress that the currently best result for the medial axis [28] is $O(n^{3+\varepsilon })$. Still, Theorem 5.3 leaves ample room for improvement, when compared to the lower bound of $\Omega (n^2 \alpha ^2(n))$ in [12]. This kindles the hope for a smaller worst-case size of straight skeletons.

Various different straight skeletons for a polytope ${\mathcal{Q}}$ can exist—at least exponentially many by the results in Sect. 4.2, even for the setting in Theorem 5.3. On the other hand, there are canonical straight skeletons for ${\mathcal{Q}}$, for example, the one obtained by resolving each vertex with the spherical skeleton [9]. The variety of possible solutions gives freedom for adapting to practical applications; we will come back to this issue in Sect. 10.

## Event Classification

The information about all possible mitered offsets and straight skeletons of a polytope is comprised in the structure of the individual events. It is therefore of interest to provide a description and categorization of these events, which is the purpose of this section. Though rich in detail and space consuming, this task leads to several insights into the geometry and combinatorics of the offsetting process, and its occasional peculiarities. Also, the presented material will be helpful when an implementation different from ours is intended. (Our algorithm is based on the largely event-independent vertex splitter in Sect. 4.2.) We supplement our study with numerous illustrations.

A problem inherent to the given input conditions is that the *initial* events can be arbitrarily complex. We therefore make no attempt to classify these events here, although this is possible at least in an overall way following the material to come, for example by referring to the vertex types.

By contrast, the *non-initial* events have a controllable structure for every input polytope ${\mathcal{Q}}$, if we limit attention to orphan-free offset surfaces. We adopt this restriction (which is commonly assumed implicitly in the literature) for the rest of this section. The anatomy of the events then can be simplified, when the polytope offset planes show a non-degenerate behavior in the following sense:

### Definition 6.1

A polytope ${\mathcal{Q}}$ fulfills the *generic condition* if there is no value $\Delta > 0$ such that 5 among its offset planes $H^{\Delta }_1, \ldots , H^{\Delta }_n$ pass through the same point. Identical offset planes (for coplanar facets of ${\mathcal{Q}}$) are counted as a single plane.

We shall see in Sect. 6.3 that coplanar facets, if not already present in ${\mathcal{Q}}$, arise naturally in the offset polytope ${\mathcal{Q}}^{\Delta }$. Definition 6.1 implies, by the geometry of planes in space, that each fixed quadruple of offset planes can share a point only for a single value of $\Delta$. Lemma 3.5 guarantees now that only offset surfaces with vertices of degree 3 are created in an event. In fact, ${\mathcal{Q}}^{\Delta }$ is a *simple polytope* for all but finitely many values of $\Delta$. Moreover, the degree of a vertex *v* to be resolved in a non-initial event is trivially limited to ${4 \atopwithdelims ()2} \cdot 2 = 12$. Actually the maximum degree is 8, by Lemma 6.4, such that the arising (degree-3) bisector graphs have at most 8 leaves, and can be singled out quickly by graph enumeration. Being especially important, the offset arrangement ${\mathcal{A}}(v)$ contains exactly 4 planes. Note that this also excludes any *multiple* non-initial event. The generic condition can be enforced for any polytope ${\mathcal{Q}}$, by infinitesimally altering the offset speeds.

Concerning 3D straight skeletons, the condition guarantees that exactly ${4 \atopwithdelims ()j}$ skeleton faces of dimension *j* are incident to each *inner* corner, for $1 \le j \le 3$. This means that a vertex *v* of ${\mathcal{Q}}^{\Delta }$, for $\Delta > 0$, can resolve into at most 4 vertices: Each of them will trace out one skeleton spoke starting at the corner *v*, and the number of spokes incident to a corner is ${4 \atopwithdelims ()1} = 4$. Corners which are *vertices* of the input polytope ${\mathcal{Q}}$ can have a larger or a smaller number of incident spokes, sheets, and cells.

There are several meaningful ways to give a taxonomy of events: By the effect they cause on the polytope, by the types of polytope faces that are interacting, or by the ‘localness’ of the events. We decided to use the last approach, as it preserves some of the features of the two-dimensional case, and also reflects the way how we implemented the detection (though not the handling) of events.

### Edge (Vanish) Events

When constructing a straight skeleton in the plane, one of the two possible (generic) events is the edge event [4]. A polygon edge shrinks to length zero, and its two neighboring edges become adjacent. Occasionally, three edges vanish at the same time, when a triangular component of the offset polygon collapses to a point.

A generalization to three dimensions entails several events of this kind. One up to six edges of the offset polytope ${\mathcal{Q}}^{\Delta }$ can vanish now at the same time, because all six edges of a tetrahedron supported by four offset planes can be present on the boundary of ${\mathcal{Q}}^{\Delta }$. We will adopt the term *edge (vanish) events*. Such events are totally local and can be detected by the fact that some edge of ${\mathcal{Q}}^{\Delta }$ attains length zero. The corresponding value of $\Delta$, where the event will (possibly) take place, can be calculated in *O*(1) time.

**Fig. 11:**
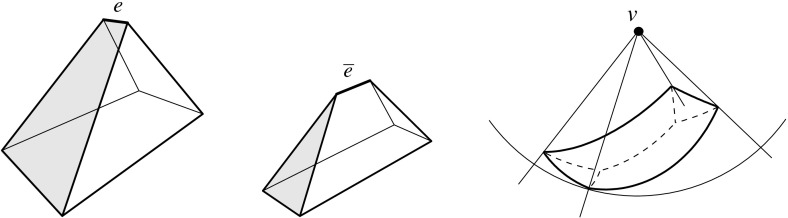
The event ${\mathcal{E}}_{1a}$. One polytope edge *e* vanishes (*left*) and flips to the edge $\overline{e}$ (*middle*). The corresponding bisector graph in the spherical quadrangle is drawn *dashed* (*right*)

We start with the simplest edge event, which we denote with ${\mathcal{E}}_{1a}$ and display in Fig. 11. (The number in the event index indicates how many edges are vanishing.) A single edge *e* disappears (left) and is replaced by the edge $\overline{e}$ which borders facets from two different offset planes (middle). In the moment when *e* ‘flips’ to $\overline{e}$, a *convex* vertex *v* of ${\mathcal{Q}}^{\Delta }$ with degree 4 is created (right). The bisector graph that resolves *v* into two vertices of degree 3 is a unique binary tree. Concerning the 3D skeleton construction, two spokes and a sheet are completed^7^ at corner *v*, and the construction of two spokes spanning a new sheet in a different bisector plane starts at *v*. Also, four already partially constructed sheets get extended further.

**Fig. 12:**
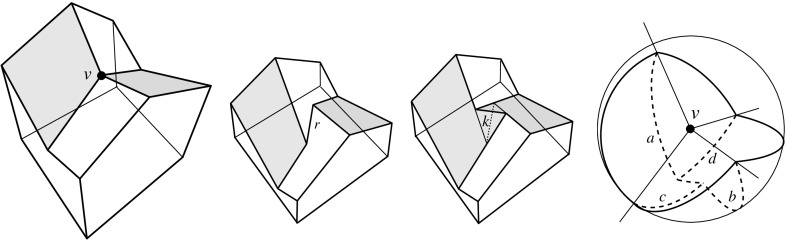
In the event ${\mathcal{E}}_{1b}$ one polytope edge vanishes and creates a saddle vertex *v*. This vertex can be resolved in two different ways, by creating either a reflex edge *r* or a convex edge *k*

The situation stays similar when the generated degree-4 vertex *v* is *pointed* but nonconvex, or is a *reflex* vertex. However, there are events where exactly one edge vanishes but with different characteristics, for instance when a *saddle vertex* is generated. Such edge events correspond to the *sticking event* in the polygon case [31], and can arise in two different forms.

See Fig. 12 for the first possibility, the event ${\mathcal{E}}_{1b}$. The created vertex *v* is incident to two convex and two reflex edges (left). Remarkably, *v* can be resolved in two different ways (middle left/right), where the vanished edge is either flipped or not. This is the example of smallest vertex degree where the offset surface is ambiguous; in fact, this is the only ambiguous event among the non-initial events (in the generic and orphan-free case). The bisector graph $G_v(\Sigma _2)$ for the latter surface $\Sigma _2$ is shown (right). The bisector graph $G_v(\Sigma _1)$ for the former surface $\Sigma _1$, which is also a tree, is obtained by prolonging the arcs *a* and *b* so as to cut off the arcs *c* and *d*. Equivalently, in the three-dimensional setting, $\Sigma _2$ is obtained from $\Sigma _1$ by adding the (single) tetrahedral cell of the offset arrangement; cf. Sect. 3.2. It is interesting to note that the spherical skeleton (see Sect. 4.2) produces the surface $\Sigma _2$, the one having more convex edges.

The actions for the 3D skeleton dictated by event ${\mathcal{E}}_{1b}$ are not unique, of course. After the completion of a sheet at *v*, the construction may continue either with a new sheet in a different, or in the *same* bisector plane. In the former instance, the skeleton continues like for a convex vertex *v*. In the latter instance, the constellation of the involved sheets and cells deserves particular attention. The completed sheet and the starting sheet are coplanar and touch at *v* after the event. The four extending sheets also have *v* as a corner, but *v* sits between two collinear spokes on their common boundary, being a ‘flat’ corner there. This is exactly the situation depicted in Fig. 9(right) in Sect. 5.2: Two of the skeleton cells incident to *v* ‘wrap around’ this corner, such that each of them is supported by both sides of a single bisector plane. These cells are therefore adjacent in two sheets. Observe that the offset polytope *retains* the incidence structure of its boundary in this event instance.

**Fig. 13:**
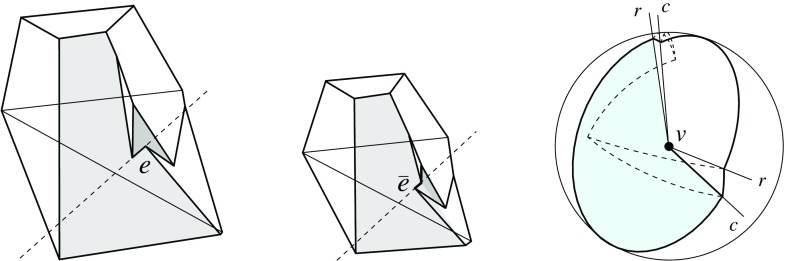
The event ${\mathcal{E}}_{1c}$ lets the polytope edge *e* vanish (*left*), and then reappear as the edge $\overline{e}$ on the same offset line (*middle*). A saddle vertex *v* with a facet having a reflex angle is created intermediately (*right*)

Figure 13 illustrates the second possibility of creating a saddle vertex *v*, which is the event ${\mathcal{E}}_{1c}$. The difference to the event ${\mathcal{E}}_{1b}$ is that *v* is incident to a facet containing a *reflex* interior angle at *v* (right). In the shown polytope, the edge *e* of the kite-shaped facet vanishes (left), and the edge $\overline{e}$ appears in the mirrored image of this facet (middle). Again we have the peculiar fact that the edges *e* and $\overline{e}$ are parallel. They do not flip, because they separate the *same* two facet offsets (shown shaded). Therefore the polytope retains its boundary incidence structure in the event, though there is a switch in the orientation of the edges *e* and $\overline{e}$. The bisector graph is a unique binary tree, that is, this event is unique. The effect of the event on the 3D skeleton is the same as in the instance with the double-adjacency above: The completed sheet and the starting sheet are coplanar, as they stem from the edges *e* and $\overline{e}$. This leads to the described geometric degeneracy in the cell structure.

We now turn to the description of edge events where more than one edge vanishes. Let us treat the case of (exactly) 3 edges next. The respective event, denoted by ${\mathcal{E}}_3$, is easy to imagine without a picture. A boundary triangle of ${\mathcal{Q}}^{\Delta }$ shrinks to a degree-3 vertex *v*, which henceforth stays a vertex of the offset polytope for a while. The bisector graph is a 3-star. Three sheets, three spokes, and also one cell of the skeleton get completed at corner *v*, and three already partially constructed sheets get adjacent at a new spoke. This spoke has *v* as a starting point.

Going up by one dimension, 6 edges vanish in the event ${\mathcal{E}}_6$ (the maximum possible) when an entire tetrahedron on the boundary of ${\mathcal{Q}}^{\Delta }$ collapses to a point *v*. Such a tetrahedron can sit on top of some facet *f* of ${\mathcal{Q}}^{\Delta }$, or constitute an isolated part such that a connected component of ${\mathcal{Q}}^{\Delta }$ disappears. The final event in the shrinking process is of the latter form. Notice that a hole of *f* may collapse in the former case. In both cases, four spokes and six sheets get completed at *v*, that is, all that can be incident to a corner in the generic case. Three cells are completed in the former case, and four in the latter. After that, *v* disappears as a vertex of ${\mathcal{Q}}^{\Delta }$ (but not as a skeleton corner, of course). The skeleton does not continue with any new sheets or spokes at *v*, though with the extension of the cell swept out by *f* in the former case. As a remarkable fact, the skeleton *spoke graph* disconnects then, at least locally: In the neighborhood of *v*, the current part of the spoke graph loses contact to the shrinking boundary of the offset polytope. Note further that the *bisector graph* is empty in both cases, because the spherical polygon is either void or a hemisphere.

**Fig. 14:**
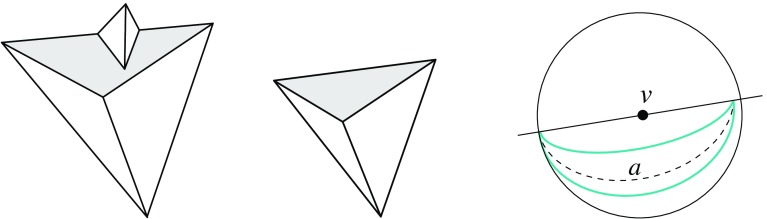
Five polytope edges vanish in the edge event ${\mathcal{E}}_5$

In a similar event, ${\mathcal{E}}_5$, exactly 5 edges may vanish when the collapsing tetrahedron has two vertices which are the endpoints of two *collinear* edges of a facet of ${\mathcal{Q}}^{\Delta }$, as in Fig. 14(left). These collinear edges merge into a single edge (middle), at the point *v* the tetrahedron shrinks into. The spherical polygon is a 2-gon in this event, formed by two semicircular arcs. The bisector graph consists of a single arc *a* (right). Four spokes, five sheets, and two skeleton cells get completed at *v*, which then disappears from ${\mathcal{Q}}^{\Delta }$. In particular, no new spokes start at *v*, that is, the spoke graph construction does not continue there again (in spite of the fact that the bisector graph is connected now). The skeleton continues with the extension of the partial sheet that corresponds to the arc *a*, and the two incident cells.

The last three events are the generalizations of the *triangle collapse* event in the polygon case. Notably, in three dimensions some edges of ${\mathcal{Q}}^{\Delta }$ can vanish also in events that *topologically* change the polytope or its non-vanishing facets. There are reasons for not including such events into the class of edge events, which will become apparent in Sects. 6.5 and 7.2. We give a brief listing of these events below, but postpone their detailed description to the subsections devoted to their classes. Detection still can be done directly, by the collapse of edges of ${\mathcal{Q}}^{\Delta }$.

An example where one edge vanishes is the event in Fig. 20(right). No new edge is produced, but an existing edge splits instead, and with it the entire polytope. Events where two edges vanish are the one in Fig. 19, and the inverses of the events in Figs. 16, 17, and 18. (Inverse events are explained in the next subsection.) Facets of ${\mathcal{Q}}^{\Delta }$ merge in these events, or holes (e)merge. There is also a case where three edges vanish, namely, the inverse of the piercing event in Sect. 6.4. A hole in a facet vanishes now, and the offset polytope may split. The event where exactly four edges vanish is the inverse of the kissing event in Sect. 6.4. As its specialities, the polytope may split and the spoke graph construction does not continue locally.

This completes the enumeration of edge events. In retrospect, these events can create all types of polytope vertices but the touching type, though only with degree at most 4. The offset polytope does not change its topology in edge events, nor does any of its facets, unless polytope pieces, facets, or holes in facets collapse entirely. The spoke graph of the skeleton locally disconnects when 5 and possibly when 6 edges vanish (in the events ${\mathcal{E}}_5$ and ${\mathcal{E}}_6$, respectively). We have also encountered an ambiguous event, the saddle vertex example ${\mathcal{E}}_{1b}$. Collinearities forced by saddle vertices have led to geometric degeneracies in the 3D straight skeleton (in ${\mathcal{E}}_{1c}$ and possibly in ${\mathcal{E}}_{1b}$).

### Inversion and Ambiguity

Before proceeding with the description of event types, let us focus on the feature that each event can be associated with its *inverse event*. One way to interpret inverse events is by considering *outer offsets*, where the offset parameter $\Delta$ decreases. For example, the inverse of the edge event in Fig. 11 transforms the polytope shown in the middle into the one on the left-hand side. The inner and the outer offset problem are equivalent, by the argument at the end of Sect. 3.2. This enables the following definition, which is also meant for the non-generic case and for non-orphan-free vertex resolution. For a vertex *v* of ${\mathcal{Q}}^{\Delta }$, let ${\mathcal{A}}'(v)$ denote the combinatorially unique offset plane arrangement for $\Delta ' < \Delta$. (Note that $\Delta '$ is negative if ${\mathcal{Q}}^{\Delta } = {\mathcal{Q}}$.)

#### Definition 6.2

Let ${\mathcal{E}}$ be an event at the polytope vertex *v* with corresponding spherical polygon ${\mathcal{S}}$. The inverse event ${\mathcal{E}}^{-1}$ of ${\mathcal{E}}$ is obtained by applying to *v* all possible bisector graphs for ${\mathcal{A}}'(v)$ inside the spherical polygon $U \setminus {\mathcal{S}}$.

The event ${\mathcal{E}}^{-1}$ gives all the valid outer offset surfaces for *v*, and thus all possible boundary structures of the polytope *before* the event ${\mathcal{E}}$, that is, when ${\mathcal{Q}}^{\Delta }$ is expanded infinitesimally. The identity $({\mathcal{E}}^{-1})^{-1} = {\mathcal{E}}$ is evident from the definition which is based on taking complements, and from the fact that ${\mathcal{A}}'(v)$ is the reflection through *v* of the arrangement ${\mathcal{A}}(v)$ in Sect. 3.

The valid offset surfaces defined by ${\mathcal{A}}'(v)$ and ${\mathcal{A}}(v)$, respectively, are combinatorially different in general. This is due to the difference of the underlying spherical polygons, which are complements but not reflections of each other. For example, the bisector graph for the event in Fig. 17 is connected, but disconnects for the inverse event into the bisector graph shown in Fig. 19. Nevertheless, inversion preserves a structural similarity that makes the ambiguity of events ‘direction-insensitive’, at least in the setting we agreed on at the beginning of Sect. 6. Let us call an event *k*-ary if exactly *k* valid and *orphan-free* inner offset surfaces exist for the respective polytope vertex *v*.

**Fig. 15:**
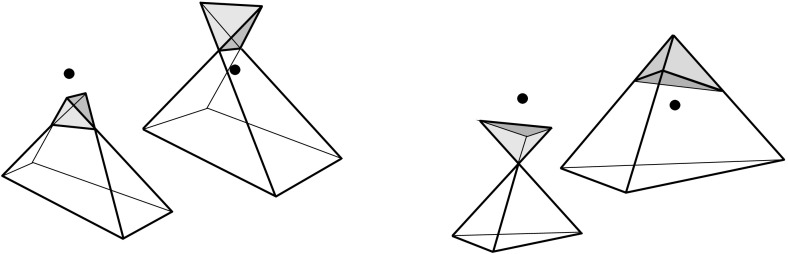
The offset surfaces for the 1-edge-vanish event ${\mathcal{E}}_{1a}$ and its inverse (*left*), and for the 3-edges-vanish event ${\mathcal{E}}_3$ and its inverse (*right*). The resolved vertex (*filled circle*) sees two tetrahedron facets for ${\mathcal{E}}_{1a}$ and ${\mathcal{E}}^{-1}_{1a}$, and one tetrahedron facet for ${\mathcal{E}}_3$ and ${\mathcal{E}}^{-1}_3$

#### Lemma 6.3

Let ${\mathcal{E}}$ be a generic non-initial event. Then ${\mathcal{E}}$ is at most 2-ary, and if ${\mathcal{E}}$ is unary (respectively, binary) then so is ${\mathcal{E}}^{-1}$.

#### Proof

The arrangement ${\mathcal{A}}(v)$ for ${\mathcal{E}}$ is defined by 4 planes, and therefore contains a single bounded cell *T* which is a tetrahedron. *T* may or may not participate in an inner offset surface for *v*. So there are at most two such surfaces, and at least one of them must be valid and orphan-free by Corollary 4.4.

The arity of events now depends on how *v* ‘sees’ the (opaque) tetrahedron *T*. Clearly, if *v* lies inside *T*, then *T* cannot influence the offset surfaces at all, and ${\mathcal{E}}$ is unary. ${\mathcal{E}}^{-1}$ is unary as well, because *v* then also lies inside the reflected tetrahedron $T'$ in ${\mathcal{A}}'(v)$. Refer to Fig. 15 now. If *v* sees a single facet of *T*, then *T* defines one or three orphan facets in the inner offset surface, and $T'$ leads to an outer offset surface which is not radially visible (or vice versa). So neither *T* nor $T'$ can be added to build a valid and orphan-free surface. Therefore both ${\mathcal{E}}$ and ${\mathcal{E}}^{-1}$ are unary. Observe next that *v* cannot see three facets of *T*, because all planes in ${\mathcal{A}}(v)$ are at the same distance from *v*. So let *v* see exactly two facets of *T*. If the presence (or the absence) of *T* defines two orphan facets in the inner surface or violates its radial visibility, then $T'$ acts the same in the outer surface. ${\mathcal{E}}$ and ${\mathcal{E}}^{-1}$ are unary in these cases. Otherwise, both *T* and $T'$ may be used or not, and ${\mathcal{E}}$ and ${\mathcal{E}}^{-1}$ are binary events. $\square$

We conjecture that the arities of ${\mathcal{E}}$ and ${\mathcal{E}}^{-1}$ are the same also in the non-generic case and for initial events. Note that *unique events* (i.e., events with only one valid inner offset surface altogether) are unary, but not conversely. For example, the inverse ${\mathcal{E}}_3^{-1}$ of the 3-edges-vanish-event ${\mathcal{E}}_3$ is unary, but has a second valid instance where the triangle which collapsed in ${\mathcal{E}}_3$ is restored as an orphan facet; see Fig. 15(right). This also shows that Lemma 6.3 cannot be extended to surfaces with orphan facets, even not when only surface vertices of degree 3 are admitted. Several unary events discussed in Sect. 6.3 also have a second inverse instance with orphan facets, though surface vertices of degree 4 or degree 6 arise there.

On the other hand, it will turn out that generic non-initial events always define unary inverse pairs $({\mathcal{E}},{\mathcal{E}}^{-1})$, with one exception: One of the (already discussed) saddle vertex events, namely ${\mathcal{E}}_{1b}$, and its inverse are binary. Consequently, four different pairs of inverse event instances exist. For two such pairs, the exchanged polytope edges are supported by offsets of the same line. The instances paired up there are *self-inverse*, because they preserve the boundary structure of ${\mathcal{Q}}^{\Delta }$. A similar situation arises in the unary saddle vertex event ${\mathcal{E}}_{1c}$ in Fig. 13.

Fixing a pair of inverse instances for resolving a vertex *v* uniquely determines the structure of the 3D straight skeleton, in the entire neighborhood of the skeleton corner *v*. A canonical way of defining such pairs is obtained, for example, by utilizing the spherical skeleton (Sect. 4.2) for vertex resolution. This may be particularly useful in the non-generic case, where most events have *k*-ary variants for $k \ge 2$, but nevertheless a canonical straight skeleton will be constructed.

We remark that, although the inverse of an event always exists, the polytopes ${\mathcal{Q}}^{\Delta - t}$ and ${\mathcal{Q}}^{\Delta + t}$ can be combinatorially (but not geometrically) different in the limit $t \rightarrow 0$. For example, when the event ${\mathcal{E}}_3$ takes place and lets a triangular facet collapse, then ${\mathcal{Q}}^{\Delta - t}$ in the limit still contains the isolated vertex *v* that results from the collapse, whereas *v* is not present in ${\mathcal{Q}}^{\Delta + t}$. Still, the inverse event ${\mathcal{E}}^{-1}_3$ is capable of restoring the vanished facet in one of its instances; see above. A similar situation arises when a tetrahedron shrinks to a vertex, in the events ${\mathcal{E}}_5$ or ${\mathcal{E}}_6$.

This implies that for any given value $\Delta > 0$, the process which shrinks ${\mathcal{Q}}$ to ${\mathcal{Q}}^{\Delta }$ can be reversed by the application of inverse events, *provided* the event history has been recorded. However, when only the information present in ${\mathcal{Q}}^{\Delta }$ is used, one will in general not end up with the initial polytope ${\mathcal{Q}}$ when expanding ${\mathcal{Q}}^{\Delta }$ again, even when all events so far have been unique: Some of the offset planes may have become redundant in the shrinking process, and then cannot be restored any more. Such a ‘loss’ of planes can only happen for edge events, and there only for ${\mathcal{E}}_3$, ${\mathcal{E}}_5$, and ${\mathcal{E}}_6$, where cells of the skeleton get completed. The inverse of a triangle collapse (where a new triangle is born), or of a tetrahedron collapse (where a new tetrahedron is born), thus do not occur in the shrinking process.

Observe finally that the inverse of each *initial* event for a polytope ${\mathcal{Q}}$ will occur as a non-inverted event, when the given input polytope has a void in the shape of ${\mathcal{Q}}$. Such events can have an arity which is exponentially high in the degree *m* of a vertex *v*. For example, we can combine $\Theta (m)$ small copies of the spherical 7-gon for the ‘binary’ vertex in Fig. 3, to construct a spherical polygon for *v* with *m* arcs. This puts an arity of $2^{\Theta (m)}$ on the event that resolves *v*.

### Facet (Boundary) Events

So-called *facet (boundary) events* are characterized by the property that either the boundaries of two coplanar facets of ${\mathcal{Q}}^{\Delta }$ get into contact, or the boundary of a facet of ${\mathcal{Q}}^{\Delta }$ gets into self-contact. A facet splits, or pinches off a hole on its boundary, or two facets or holes merge. Facet events can be viewed as counterparts of the split event for planar straight skeletons [4], where the boundary of the input polygon self-contacts and the polygon splits apart.

Detection of events in this class can still be done locally, either directly by observing the collapse of edges in ${\mathcal{Q}}^{\Delta }$, or by looking at the self-contacting facet *f* of ${\mathcal{Q}}^{\Delta }$. The value of $\Delta$ where the boundary of *f* (possibly) gets into self-contact can be calculated directly in $O(k^2)$ time, when *f* has *k* edges. As an alternative, a weighted straight skeleton for *f* can be used. Negative edge weights [13] may occur now, however, because *f* can have edges that shift toward its exterior. A simple and usually fast implementation is by triangulating all the facets of ${\mathcal{Q}}$ beforehand, and detecting facet changes by the collapse of triangles [3]. This works in *O*(*h*) overall time for all facets per increase of $\Delta$, when ${\mathcal{Q}}^{\Delta }$ has *h* facets and each of them is of constant size.

**Fig. 16:**
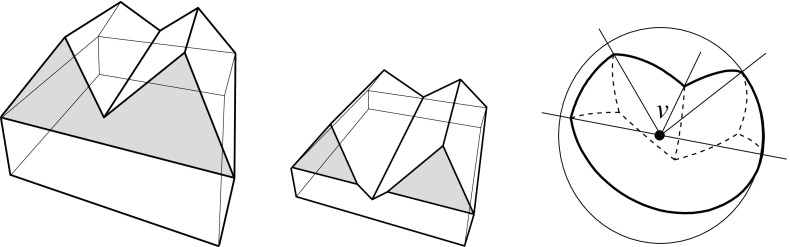
In the facet event ${\mathcal{F}}_{\textsc {i}}$, the *shaded facet* splits in a vertex/edge contact

**Fig. 17:**
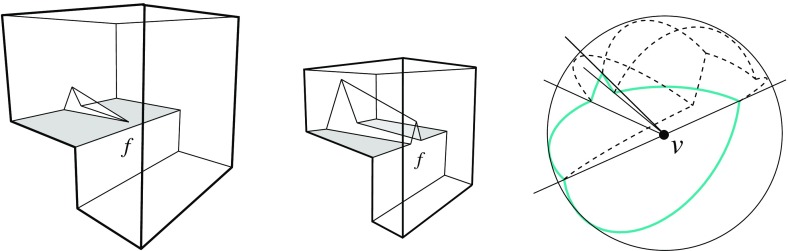
The vertex/edge contact ${\mathcal{F}}_{\textsc {ii}}$ is similar to the facet event in Fig. 16

Facets events show the richest forms, and they can be systematized by the dimension of the facet boundary pieces that get into contact. Most similar to the planar case is the type *vertex*/*edge contact*, ${\mathcal{F}}_{\textsc {i}}$, which is illustrated in Fig. 16: A vertex of the shaded facet runs into an opposite edge. A degree-5 vertex *v* of ${\mathcal{Q}}^{\Delta }$ is created by this contact, and the shaded facet disconnects at *v*. Observe that *v* is non-touching, but is neither a pointed vertex nor a saddle vertex. We may call *v* an *almost pointed* vertex, because its spherical polygon ${\mathcal{S}}$ is contained in a closed hemisphere of *U*, but not in any open one (as is required for being pointed); ${\mathcal{S}}$ is a pentagon where one arc is a semicircle. The bisector graph is a binary tree with 3 inner nodes, which reflects that *v* gets split into three degree-3 polytope vertices.

The same anatomy arises for a similar case of a vertex/edge contact, ${\mathcal{F}}_{\textsc {ii}}$, displayed in Fig. 17. In particular, the bisector graph has the same combinatorial tree structure in both cases, and is unique such that there exists only one valid offset surface. Any other surface built from the same planes necessarily contains facets offsetting toward the exterior of the polytope (cf. Lemma 3.2). Notice that—in contrast to before—the vertical facet *f* *extends* now locally at *v* after the event. In the 3D skeleton, a spoke ends at the corner *v* and three spokes start there. Also, four sheets are extended, and two sheets are created.

**Fig. 18:**
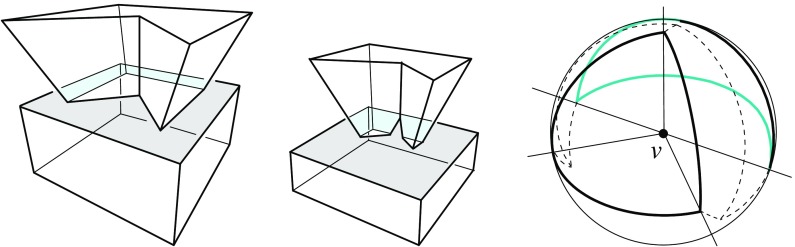
The facet event ${\mathcal{F}}_{\textsc {iii}}$ produces a tunnel in the vertex/edge contact

There is another case of a vertex/edge contact, the facet event ${\mathcal{F}}_{\textsc {iii}}$ where the offset polytope alters its topology. This is illustrated in Fig. 18. The shaded facet contains a hole (left), and its boundary gets in self-contact there, squeezing the hole into two (middle). A vertex *v* of degree 5 is created intermediately (right), which is of the touching type now: The boundary of the spherical polygon consists of two parts, a triangle and a 2-gon. On the other hand, the spherical polygon is still connected, and the bisector graph even has the same tree structure as in the events ${\mathcal{F}}_{\textsc {i}}$ and ${\mathcal{F}}_{\textsc {ii}}$. This is interesting in view of the fact that a tunnel is created in the offset polytope by this event. The actions for the skeleton are the same as above.

**Fig. 19:**
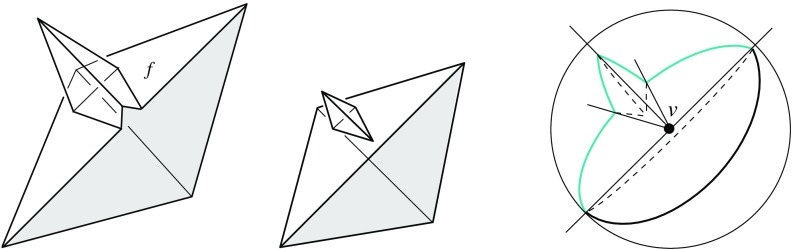
The facet event ${\mathcal{F}}_{\textsc {iv}}$ gives rise to a vertex/vertex contact

Consider Fig. 19 next. The geometry of the shown polytope (which has similarities to the polytope for the event ${\mathcal{E}}_5$ in Fig. 14) generically enforces a facet event, ${\mathcal{F}}_{\textsc {iv}}$, of the type *vertex*/*vertex contact*. The two short edges of the shaded facet simultaneously shrink to length zero (left). At the same moment, the object on top of the adjacent facet, *f*, shrinks so as to touch the boundary of *f* at a vertex *v* of ${\mathcal{Q}}^{\Delta }$, where two collinear edges merge now into one. The objects then drifts away on top of the generated hole of *f* (middle). The vertex *v* has degree 5 and is of the almost-pointed type. The spherical polygon is connected, but the bisector graph is disconnected, being a forest consisting of a 3-star and a single arc. This graph has only one inner node; accordingly, *v* is ‘resolved’ into only one vertex of the offset polytope. That is, *v* does not split at all, but rather loses two collinear edges.

Actually, this event is the inverse of the facet event ${\mathcal{F}}_{\textsc {ii}}$ in Fig. 17, but in the *complementary setting* (i.e., where the interior and exterior of the polytope are exchanged). Therefore, the respective reverse actions take place in the skeleton. Note at this point that the inverse of a facet event has to be a facet event again.

**Fig. 20:**
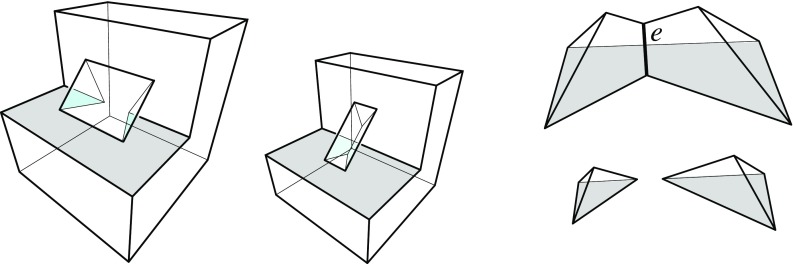
The vertex/vertex contact ${\mathcal{F}}_{\textsc {v}}$ shows different characteristics (*left*/*middle*). Its inverse in the complementary setting (*right*) is the facet event ${\mathcal{F}}_{\textsc {vii}}$, which splits the polytope

We also observe that the two contacting vertices have to move along the same offset line. Another case where this happens is depicted in Fig. 20. The characteristics of this event, ${\mathcal{F}}_{\textsc {v}}$, differ from the preceding one. Two vertices at the far side of the shaded facet meet (left), such that a hole pinches off from the boundary of this facet, and another one for the adjacent facet (middle). At the same moment, a touching vertex *v* of ${\mathcal{Q}}^{\Delta }$ of degree 6 is generated. Vertex *v* resolves into only two offset vertices, because two collinear edges of the shaded facet merge into one. Thereby, a tunnel is generated in the offset polytope. The spherical polygon is connected but contains two holes (similar as in Fig. 18). The bisector graph disconnects, however, into a single arc and a binary tree with two inner nodes. In the skeleton, two spokes arrive at corner *v*, and two are created. Five sheets continue, and one sheet is born.

**Fig. 21:**
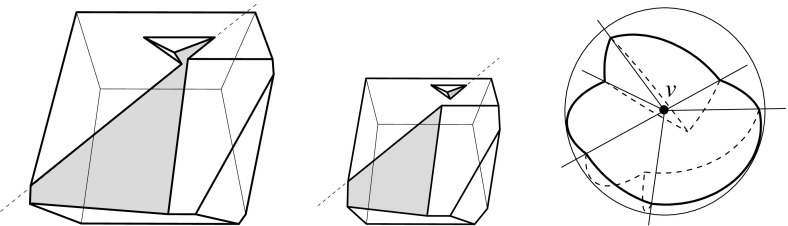
A saddle vertex is created in the vertex/vertex contact ${\mathcal{F}}_{\textsc {vi}}$. The *dashed straight line* (*left* and *middle*) indicates the offset line that the contacting vertices are moving on

The last distinguishable case of a vertex/vertex contact is when a saddle vertex of ${\mathcal{Q}}^{\Delta }$ is generated, in the facet event ${\mathcal{F}}_{\textsc {vi}}$. See Fig. 21. The shaded facet splits, and a hole is released from the boundary of an adjacent facet, with a small polytope on its top. The created saddle vertex *v* is of degree 6 with two collinear edges, and the spherical polygon is a hexagon. The bisector graph is a forest with two 3-stars, and *v* resolves accordingly into two vertices. Again, two skeleton spokes arrive at corner *v*, and two are born. However, six sheets arrive at *v* and all six extend. Interestingly, the degenerate incidence structure shown in Fig. 9(right) occurs again, because the two sheets that stem from the collinear edges of *v* are coplanar. Observe that the contacting vertices do not span a polytope edge, nor do the new offset vertices after *v*’s resolution. Consequently, a double-adjacency of cells arises.

Finally, there also exist facet events of mixed type, and a *vertex*/*vertex*/*edge* contact may occur. This type, ${\mathcal{F}}_{\textsc {vii}}$, is shown in Fig. 20(right). One may be tempted to classify this event (and, similarly, also ${\mathcal{F}}_{\textsc {iv}}$) as an *edge event*, as it causes some edge *e* to vanish. However, the shaded facet splits at the created vertex *v* of ${\mathcal{Q}}^{\Delta }$, and with it another facet, which settles the type of the event. Actually, this event represents the ‘pure’ generalization to three dimensions of the planar *split event*, because it splits the polytope at *v*, at least locally. Note that *v* is a touching vertex of degree 6, and resolves into only two vertices. The bisector graph and the spherical polygon disconnect as well (but not the spoke graph), into two 3-stars and two spherical triangles, respectively. This event is the inverse of the vertex/vertex contact event ${\mathcal{F}}_{\textsc {v}}$, in the complementary setting. In the skeleton, the respective reverse actions take place.

**Fig. 22:**
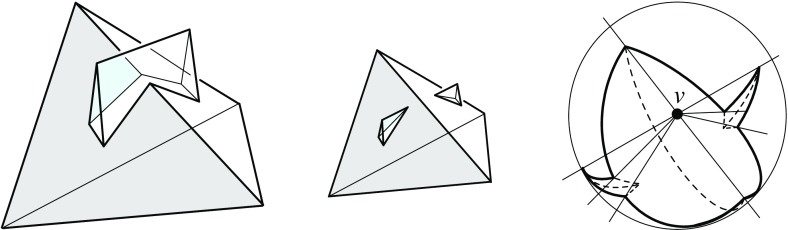
The facet event ${\mathcal{F}}_{\textsc {viii}}$ with a vertex/edge/edge contact

A facet event of the mixed type can also involve a *vertex*/*edge*/*edge* contact. Such an event, ${\mathcal{F}}_{\textsc {viii}}$, is depicted in Fig. 22. The shaded facet pinches off a hole as does its mirrored neighbor facet, when two of their common vertices meet at vertex *v* of ${\mathcal{Q}}^{\Delta }$ (left). This creates an edge of ${\mathcal{Q}}^{\Delta }$ by merging, but another edge arrives there at the same time and splits. Consequently, *v* is of degree 8 and has two pairs of collinear edges (right). The degree of the constructed skeleton corner *v* is still 4 (as in all other non-initial events), because *v* splits into only two vertices in the offset polytope (middle). These vertices belong to two objects sitting on the two holes born in the event. The spherical polygon is boundary-connected, but the bisector graph is a forest with three components, two 3-stars and an isolated arc. Once more, two skeleton spokes get completed and two created. Six already existing sheets concur at *v* and extend, like in the event ${\mathcal{F}}_{\textsc {vi}}$. But now *v* is of the almost-pointed type in ${\mathcal{Q}}^{\Delta }$, and its degree is the maximum that can be attained in the generic case:

#### Lemma 6.4

For all $\Delta > 0$, the degree of the vertices of ${\mathcal{Q}}^{\Delta }$ is limited to 8.

#### Proof

Let *v* be some vertex of ${\mathcal{Q}}^{\Delta }$ for given $\Delta > 0$. The number of offset planes passing through *v* is at most 4, and each plane can contribute at most 2 facets of ${\mathcal{Q}}^{\Delta }$ incident to *v*. But the number of facets and edges incident to *v* must be the same, because these faces correspond bijectively to the arcs and nodes of the spherical polygon for *v*.$\square$

To convince ourselves that the enumeration of facet event types is complete, we observe that the *vertex*/*vertex*/*edge*/*edge* contact has already been covered by the event ${\mathcal{F}}_{\textsc {viii}}$. Moreover, the concurrence of three non-neighboring vertices of ${\mathcal{Q}}^{\Delta }$, or of three edges, respectively, is outruled by the generic condition in Definition 6.1: At least five offset planes would have to pass through a common point, as can be seen from the discussion of collision types in the next subsection.

In summary, all facet events are unary, as are their inverses by Lemma 6.3. Notice that the latter mostly stem from coplanar constellations (e.g., ${\mathcal{F}}^{-1}_{\textsc {i}}$, ${\mathcal{F}}^{-1}_{\textsc {vi}}$, and ${\mathcal{F}}^{-1}_{\textsc {viii}}$) but are still generic. Some facet events cause a topological change of the offset polytope. However, facet events cannot disconnect the spoke graph: The singular vertex created on the facet boundary cannot disappear without giving rise to new offset vertices. Forced collinearities (in the event ${\mathcal{F}}_{\textsc {vi}}$) may lead to a degenerate geometry of straight skeleton cells, as with edge events, but not to deficiencies in the spoke graph.

### Global (Collision) Events

It remains now to consider the class of *global (collision) events*, where combinatorially unrelated faces of ${\mathcal{Q}}^{\Delta }$ collide. By the global nature of such events, they can be recognized only by inspecting the entire polytope boundary, unless an auxiliary data structure is used. In an implementation, one might want to maintain a suitable volume partition of ${\mathcal{Q}}^{\Delta }$, for example a tetrahedrization based on Steiner points in the interior of ${\mathcal{Q}}^{\Delta }$; see e.g. [14]. Events then can be identified by keeping track of tetrahedra collapses in the partition, similar as in one dimension lower for the facet events, whose detection is then automatically included.

By convention, we assign the inverse of a global event to the same class. All such events are *unique*, because the resolved vertex *v* always lies inside the tetrahedron *T* defined by the arrangement ${\mathcal{A}}(v)$; see the proof of Lemma 6.3. For the same reason, global events cause a topological change of ${\mathcal{Q}}^{\Delta }$, and the boundary of the spherical polygon is necessarily disconnected. As an interesting fact, the bisector graph has to contain cycles now. Again, the distinction of event types can be done by the dimension of the polytope faces that are interacting.

**Fig. 23:**
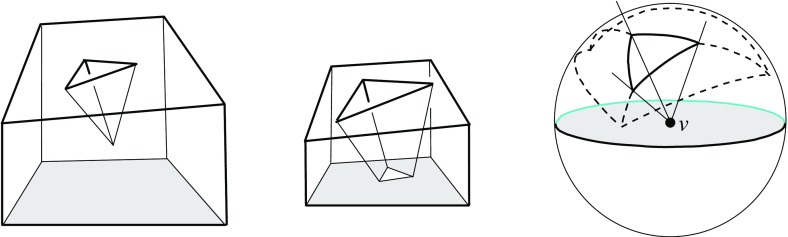
In the piercing event, ${\mathcal{G}}_{\textsc {p}}$, a polytope vertex touches the interior of a facet

**Fig. 24:**
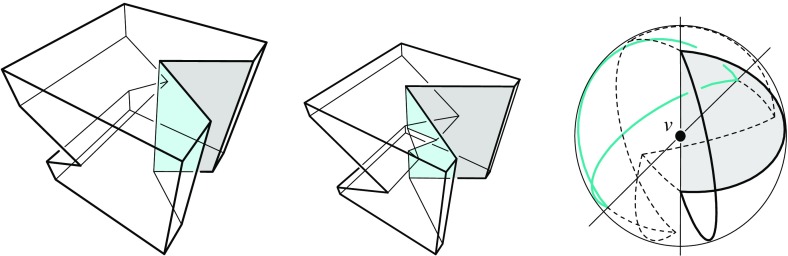
In the kissing event, ${\mathcal{G}}_{\textsc {k}}$, two polytope edges contact at a point in their interiors

See Fig. 23 for the first type, the *vertex*/*facet collision*. The front vertex of a tetrahedral dent of the polytope approaches the shaded facet (left) and pierces a triangular hole into its interior (middle), which suggests the name *piercing event*, ${\mathcal{G}}_{\textsc {p}}$, for short. In between, a touching vertex *v* of ${\mathcal{Q}}^{\Delta }$ is generated (right), which is incident to three facets, and touches the interior of one facet. The spherical polygon is connected but contains a hole. Its boundary consists of a spherical triangle and a great circle, the latter stemming from the touched facet. The bisector graph is a cycle of length 3, connected to its leaves in the spherical triangle. Conforming to this graph structure, the vertex *v* resolves into 3 vertices that span a hole of the pierced facet. This constructs a tunnel in the offset polytope. In the 3D skeleton, three sheets get *v* as a corner and extend, but their common spoke is completed there. Three spokes are born at *v*, along with three sheets spanned by them.

The inverse of the piercing event, ${\mathcal{G}}^{-1}_{\textsc {p}}$, lets 3 edges of the offset polytope vanish. However, a tunnel disappears or (in the complementary setting) the offset polytope splits locally—the reason why we classify ${\mathcal{G}}^{-1}_{\textsc {p}}$ as a global event and not as an edge event. Still, ${\mathcal{G}}^{-1}_{\textsc {p}}$ can be detected directly, like the inverse of the following event.

The *edge*/*edge collision* or *kissing event*, ${\mathcal{G}}_{\textsc {k}}$, has a similar anatomy that is displayed in Fig. 24. The front edges of two reflex wedges of the polytope approach each other (left). In the moment they contact at a point in their interiors, a touching vertex *v* of ${\mathcal{Q}}^{\Delta }$ of degree 4 is created, incident to two pairs of collinear edges (right). The vertex *v* then resolves into 4 vertices spanning a nonplanar quadrangle (middle), which opens a tunnel in the offset polytope. We recall from the beginning of Sect. 6 that 4 new offset vertices is the maximum that can be achieved under generic conditions. The bisector graph consists of a 4-cycle connected to the spherical polygon boundary, which disconnects into two semicircular 2-gons. The spherical polygon itself is connected. Interestingly, in the 3D skeleton, an *isolated* corner *v* with four incident spokes is generated. Four sheets are initiated too, and the two sheets traced out by the front edges of the colliding wedges are extended. This leads to a (temporarily) isolated part of the spoke graph. However, this part stays connected to the offset polytope boundary from the exterior, and will therefore connect to some other part of the spoke graph in later events.

The inverse of the kissing event, ${\mathcal{G}}^{-1}_{\textsc {k}}$, is the only event where exactly 4 edges of the offset polytope vanish. But the topological effect of this event is the same as for the inverse piercing event, which hinders its inclusion into the class of edge events.

Combinatorially, there are six possibilities to pair up polytope faces of dimensions 0 to 2. Let us go over them systematically now. Two possibilities are (partially) ruled out by our generic assumption: The type *vertex*/*vertex collision* leads to 6 or 5 offset planes passing through the same point, unless *two* pairs of identical planes are present. In that case, the event shapes into the facet event ${\mathcal{F}}_{\textsc {v}}$ in Fig. 20(left). Similarly, a *vertex*/*edge collision* yields 5 concurring offset planes, unless an identical pair is involved. The resulting event is the facet event ${\mathcal{F}}_{\textsc {iii}}$ in Fig. 18, but in the complementary setting. The *vertex*/*facet collision* is the piercing event ${\mathcal{G}}_{\textsc {p}}$.

By contrast, the type *edge*/*edge collision* is only partially covered by the kissing event, because the contacting edges may be collinear. One or two pairs of coinciding offset planes are involved then, but in the latter case the planes have differently-oriented normal vectors in each pair. Such a collinear contact of edges, and also the next type, the *edge*/*facet collision*, can be interpreted as limit cases of the type *facet*/*facet collision*, the last possible combination. This event necessarily requires two coinciding offset planes of different orientation.

These last three event types contain geometric degeneracies which are *not* excluded by the generic assumption. We observe that, in the moment of collision, more than one new vertex can be created in the polytope ${\mathcal{Q}}^{\Delta }$. Still, such an event is not a multiple event, as it does not take place at a single point in space. Rather, it can be seen and treated as a collection of simultaneous events: Each new vertex *v* of ${\mathcal{Q}}^{\Delta }$ can be be resolved individually, and the results assembled into the surface of the offset polytope. The individual events are simple modifications of the piercing event and the kissing event, respectively, and therefore are unique. Consequently, the combined event is unique as well. We do not further elaborate on the details here.

### Concluding Observations

The enumeration of the generic events is now complete. In spite of their sometimes quite complex anatomy, all events but one are unary. In the less obvious cases, this can be verified easily by using bisector graphs as a tool. Another general observation is that events do not change class when being inverted (which is partially due to the convention made for global events).

One can give a gross classification of events, based on whether or not the offset polytope ${\mathcal{Q}}^{\Delta }$ or any of its non-vanishing facets experiences a boundary contact (or loses such a one). For edge events this does not happen, whereas all the facet events and global events involve an effect of that kind. This suggests to join the latter events into the overall class of *contacting* events, as opposed to the *contact-free* edge events. This distinction will turn out important in Sect. 7.2. We recall that contacting events can neither complete the construction of a cell in the straight skeleton, nor disconnect its spoke graph.

On the other hand, only the contacting events have the ability to alter the 3D topology of the polytope. Tunnels may be created or destroyed, either locally like in the facet event ${\mathcal{F}}_{\textsc {iii}}$ in Fig. 18 and its inverse, respectively, or globally like in the piercing event ${\mathcal{G}}_{\textsc {p}}$ and its inverse. Similarly, when the polytope splits, as for example in the event ${\mathcal{F}}_{\textsc {vii}}$ in Fig. 20(right), then it does so either locally by destroying a tunnel, or globally by incrementing the number of its connected components. The latter change causes the polytope boundary to disconnect. As a remarkable fact—which is already reflected in our event enumeration—this is the only way a boundary disconnection can happen, also in the non-generic case:

#### Lemma 6.5

Let ${\mathcal{Q}}$ be an arbitrary polytope. In a valid offsetting process (according to Definition 3.1) where ${\mathcal{Q}}$ shrinks to volume zero, no new voids can appear in any offset polytope.

#### Proof

Assuming the contrary, let a corresponding event happen at the point *v* in the offset polytope ${\mathcal{Q}}^{\Delta }$, for some value $\Delta$. Denote with *X* the new void in the polytope ${\mathcal{Q}}^{\Delta + \varepsilon }$, for sufficiently small $\varepsilon$. If point *v* lies on the boundary of ${\mathcal{Q}}^{\Delta }$ such that the void was released from there, then there exists a ray emanating from *v* which intersects the boundary of *X* at least twice. This contradicts the radial visibility of the created offset surface. Otherwise, the void did appear in the interior of ${\mathcal{Q}}^{\Delta }$. By the nature of the shrinking process, we must have $v \in X$, and the offset planes supporting the facets of *X* have collectively swept over *X* during the interval $[\Delta ,\Delta + \varepsilon ]$, starting from *v*. But prior to the resolution of *v*, these planes must have arrived at *v*. Therefore, they have swept over points in *X* already earlier, for offset values smaller than $\Delta$. This cannot happen in a valid offsetting process. $\square$

Notice that, nevertheless, an event capable of creating a void in the *interior* of ${\mathcal{Q}}^{\Delta }$ exists, namely, the inverse ${\mathcal{E}}^{-1}_6$ of the 6-edges-vanish event ${\mathcal{E}}_6$ where a tetrahedron collapses. By Lemma 6.5, the event ${\mathcal{E}}^{-1}_6$ does not occur in the shrinking process. (Compare, in this context, the observations at the end of Sect. 6.2.) An event where a void is released from the *boundary* of ${\mathcal{Q}}^{\Delta }$ is generally missing, because it presumes an invalid shape of the offset surface.

This situation parallels the two-dimensional case. When the input polygon is shrunk, no holes can be generated, neither in its interior nor at its boundary. It is instructive to visualize that such planar hole generations do take place for the polytope facets—in the piercing event and, for example, in the facet events of type vertex/vertex contact, respectively. The intuitive explanation for the lack of the corresponding events in the *input dimension* 2 (for a polygon) and 3 (for a polytope) is that an additional dimension is needed to enable the generating boundary singularities.

**Table:** 

Event	Fig	Contact	Deg *v*	*v*-type	Bi-graph	${\mathcal{S}}$	Completed	Created	Spo-graph	Topol	Inv
${\mathcal{E}}_{1a}$	6.1	Free	4	Convex	$B_4$	b-c	2/1/0	2/1/0	c	–	$\star$
${\mathcal{E}}_{1b}$	6.2	Free	4	Saddle	$B_4$	b-c	2/1/0	2/1/0	c	–	$\star$
${\mathcal{E}}_{1c}$	6.3	Free	4	Saddle	$B_4$	b-c	2/1/0	2/1/0	c	–	$\star$
${\mathcal{E}}_3$	–	Free	3	Convex	$B_3$	b-c	3/3/1	1/0/0	c	–	$\notin$
${\mathcal{E}}_5$	6.4	Free	2	Flat	$B_2$	b-c	4/5/2	0/0/0	n	–	$\notin$
${\mathcal{E}}_6$	–	Free	0	Flat	$\emptyset$	b-c	4/6/3-4	0/0/0	n	–	$\notin$
${\mathcal{F}}_{\textsc {i}}$	6.6	v/e	5	a-pointed	$B_5$	b-c	1/0/0	3/2/0	c	–	$\star$
${\mathcal{F}}_{\textsc {ii}}$	6.7	v/e	5	a-pointed	$B_5$	b-c	1/0/0	3/2/0	c	–	$\overline{{\mathcal{F}}_{\textsc {iv}}}$
${\mathcal{F}}_{\textsc {iii}}$	6.8	v/e	5	Touching	$B_5$	b-d	1/0/0	3/2/0	c	Tunnel	$\star$
${\mathcal{F}}_{\textsc {iv}}$	6.9	v/v	5	a-pointed	$B_2$ $B_3$	b-c	3/2/0	1/0/0	c	–	$\overline{{\mathcal{F}}_{\textsc {ii}}}$
${\mathcal{F}}_{\textsc {v}}$	6.10$\ell$	v/v	6	Touching	$B_2$ $B_4$	b-d	2/0/0	2/1/0	c	Tunnel	$\overline{{\mathcal{F}}_{\textsc {vii}}}$
${\mathcal{F}}_{\textsc {vi}}$	6.11	v/v	6	Saddle	$B_3$ $B_3$	b-c	2/0/0	2/0/0	c	–	$\star$
${\mathcal{F}}_{\textsc {vii}}$	6.10*r*	v/v/e	6	Touching	$B_3$ $B_3$	d	2/1/0	2/0/0	c	Split	$\overline{{\mathcal{F}}_{\textsc {v}}}$
${\mathcal{F}}_{\textsc {viii}}$	6.12	v/e/e	8	a-pointed	$B_2$ $B_3$ $B_3$	b-c	2/0/0	2/0/0	c	–	$\star$
${\mathcal{G}}_{\textsc {p}}$	6.13	v/f	3	Touching	$C_3$	b-d	1/0/0	3/3/0	c	Tunnel	$\star$
${\mathcal{G}}_{\textsc {k}}$	6.14	e/e	4	Touching	$C_4$	b-d	0/0/0	4/4/0	s	Tunnel	$\star$

The variety of the skeleton construction events and their properties is admittedly confusing, and an overview may be helpful. The following table summarizes the (generic and orphan-free) events that can take place in the shrinking process for a nonconvex polytope. We respect the order of classes and the notations established in the preceding subsections: edge events ${\mathcal{E}}_x$, facet events ${\mathcal{F}}_x$, and global events ${\mathcal{G}}_x$. For each event, the following information is displayed in the respective table row:

The generating type of *contact*, if any (indicated as **v**ertex/**e**dge/**f**acet); the *degree* and the *type* of the offset polytope vertex *v* created at this moment; the structure of the *bisector graph* (we denote with $B_k$ a binary tree with *k* leaves, and with $C_k$ a length-*k* cycle connected to *k* leaves); the connectivity behavior of the *spherical polygon* ${\mathcal{S}}$ (**b**oundary-**c**onnected, **b**oundary-**d**isconnected, or **d**isconnected); the number of spokes/sheets/cells *completed* and *created* in the straight skeleton being constructed (the number of *extending* skeleton faces is the rest to 4 / 6 / 4); the local behavior in the *spoke graph* construction (**c**ontinuing, **n**ot continuing, or **s**tarting); the *3D topological changes* in the offset polytope, if any; and finally the *inverse event* (here $\overline{{\mathcal{F}}}$ denotes the event ${\mathcal{F}}$ in the complementary setting, ‘$\star$’ indicates that the inverse event only relates to itself, and $\notin$ means that the event exists but does not arise). The *arity* of events is not included; we refer to the beginning of this subsection.

Global events of the collision types collinear-edge/edge, edge/facet, and facet/facet are not listed; these simultaneous events can have a more complex structure. Apart from that, the table represents a complete enumeration, as is also indicated by the regularities of vertex degrees (Column 4) and of bisector graph structures (Column 6).

## Roof Complexes

Our main results on mitered offset surfaces and straight skeletons are based on arrangements of planes in 3-space, and therefore can be generalized naturally. We will address extensions in three different directions—which can be combined—in the present section and in Sects. 8 and 9, respectively. Arrangements will play a double role, for resolving vertices of a polytope ${\mathcal{Q}}$ on the one hand, and for defining piecewise-linear surfaces above ${\mathcal{Q}}$, on the other.

It is well known that the straight skeleton of a polygon in the plane defines a *roof surface* in three dimensions [4, 21]. We have mentioned this property already in Sect. 4.1 and illustrated it (for the weighted case) in Fig. 7. In short terms, the straight skeleton is the vertical projection of the roof surface. Similar is the situation in one dimension higher, which we will exploit now to define more general cell decompositions for polytopes.

### Basic Properties

Let ${\mathcal{Q}}$ be our polytope in ${\mathbbm {R}}^3$. Every straight skeleton, ${{\mathcal {SK}}}$, of ${\mathcal{Q}}$ defines a four-dimensional surface as follows. Let $C_1, \ldots , C_n$ be the cells that constitute ${{\mathcal {SK}}}$, and denote with $H_i$ the plane that supports the cell $C_i$ at a facet of ${\mathcal{Q}}$. We identify ${\mathbbm {R}}^3$ with the hyperplane $W^0: t = 0$ of ${\mathbbm {R}}^4$, where *t* stands for the 4th coordinate. Now consider the hyperplanes

$$\begin{aligned} L_i = \{ (x,t) \in {\mathbbm {R}}^4 \mid t = \delta (x,H_i) \} \end{aligned}$$

with $\delta (x,H_i)$ measuring the normal distance of a point $x \in W^0$ to $H_i$ (signed to be positive on the side of $H_i$ that contains $C_i$). Viewing $L_i$ as a linear function on $W^0$, the *skeleton roof* for ${{\mathcal {SK}}}$ is defined as the piecewise-linear and continuous function $\varphi : {\mathcal{Q}}\rightarrow {\mathbbm {R}}$ with $\varphi (x)= L_i(x)$ for all $x \in C_i$ and $i = 1, . . . , n$.

We can also define more general roofs for ${\mathcal{Q}}$, by relaxing the condition that binds roofs to straight skeletons.

#### Definition 7.1

Let $\psi : {\mathcal{Q}}\rightarrow {\mathbbm {R}}$ by a continuous function with $\psi (x)= 0$ for all *x* on ${\mathcal{Q}}$’s boundary, and $\psi (x)= L_i(x)$ for at least one index *i*. We call the graph of $\psi$ a *roof* for the polytope ${\mathcal{Q}}$, and the subset of ${\mathcal{Q}}$ where $\psi$ is non-differentiable a *roof complex* for ${\mathcal{Q}}$.

Roof complexes are cell complexes inside ${\mathcal{Q}}$, coming from vertically projecting onto $W^0$ the faces of the piecewise-linear surface in ${\mathbbm {R}}^4$ defined by a roof. Among such decompositions of ${\mathcal{Q}}$, there have to be all its straight skeletons. But also different cell complexes are contained in this class, as is indicated (in one dimension lower) by Figs. 4 and 8. However, the local incidence structure in a roof complex has to be the same as in a straight skeleton, as both structures are defined by the same type of piecewise-linear functions.

Note the similarity between roof surfaces and *offset surfaces* in Definition 3.1. In fact, offset surfaces can be seen as the roofs defined by a radial function $\psi _U$ on a sphere *U* in ${\mathbbm {R}}^3$. Roof complexes on *U* then have *bisector graphs* as their spoke graphs, and among them the *spherical skeleton* [9] takes the role of the skeleton roof complex. This reflects the dimension-recursive structure of straight skeletons, which we will make use of in Sects. 7.3 and 9.

One difference to straight skeletons is that a roof complex can contain additional cells, namely, more than one connected cell for a given facet $f_i$ of ${\mathcal{Q}}$. In particular, if there exist orphan cells for $f_i$, then they are not necessarily concatenated by corners of the complex like in a straight skeleton; see Sect. 5.2. Only one of these cells is bordered by $f_i$. Nevertheless, the cells have a useful property even for roof complexes which are not orphan-free.

#### Lemma 7.2

The union of cells in a roof complex for ${\mathcal{Q}}$, defined by a fixed facet $f_i$ of ${\mathcal{Q}}$, is a point set which is monotone with respect to $f_i$.

#### Proof

Let *X* be the point set in question, and let $\psi$ be the function that induces the roof complex for ${\mathcal{Q}}$. We generalize the proof in [3] to one more dimension. Take some line $\ell$ in the hyperplane $W^0$ and normal to the supporting plane $H_i \supset f_i$, and consider the restriction $\psi |_{\ell }$ of $\psi$ to $\ell$. We have $\psi |_{\ell }(x) = L_i(x)$ if $x \in X$, and $\psi |_{\ell }(x) = L_j(x) < L_i(x)$ for some index $j \ne i$ if $x \in {\mathcal{Q}}\setminus X$, the inequality coming from the fact that $\ell$ is normal to the plane $H_i$ but not to the plane $H_j$. As a consequence, if *X* is not monotone such that $\ell$ can be chosen to intersect *X* in a disconnected set, then the function $\psi |_{\ell }$ is discontinuous. But this is a contradiction, because $\psi |_{\ell }$ is the restriction of the continuous function $\psi$. $\square$

By the monotonicity property expressed in Lemma 7.2, no voids can exist in the cells of a roof complex, even if ${\mathcal{Q}}$ itself has voids. This has the nice effect that the proof of Lemma 5.2 in Sect. 5 generalizes, which is somewhat unexpected as roof complexes are a much more general concept of cell complexes.

#### Lemma 7.3

The spoke graph of any roof complex for ${\mathcal{Q}}$ does not contain parts which are isolated from ${\mathcal{Q}}$’s boundary, and therefore has *O*(*n*) connected components.

Although not every roof corresponds to a straight skeleton, any fixed roof ${\mathcal{R}}$ for ${\mathcal{Q}}$ encodes a unique shrinking process for ${\mathcal{Q}}$. This has been discussed for the two-dimensional case in [4]. Let $W^{\Delta }$ stand for the horizontal hyperplane $t = \Delta$ of ${\mathbbm {R}}^4$, for $\Delta \ge 0$. ($W^{\Delta }$ represents the ‘water level’ in the two-dimensional island model.) Consider the intersection $W^{\Delta } \cap {\mathcal{R}}$. This intersection bounds a polytope ${\mathcal{Q}}^{\Delta }$, which shrinks for increasing $\Delta$. As all roof hyperplanes have the same slope, the edges and vertices of ${\mathcal{Q}}^{\Delta }$ move in angle bisector planes, and along trisector lines, respectively. Clearly, this offsetting process traces out the roof complex for ${\mathcal{Q}}$ that corresponds to ${\mathcal{R}}$. Notice that the process respects Definition 3.1, because a valid cell complex (without overlapping parts) is constructed. If ${\mathcal{R}}$ is a skeleton roof, then a particular straight skeleton construction process for ${\mathcal{Q}}$ results, as in Sect. 5.

**Fig. 25:**
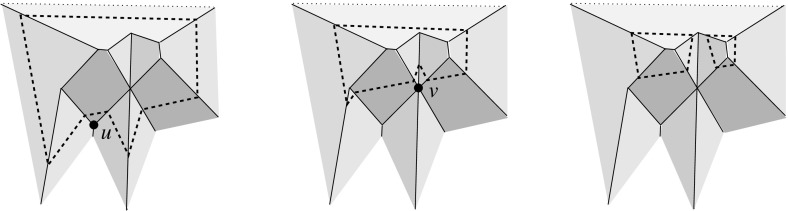
Roof-based shrinking process for a polygon. Interesting events arise at vertices *u* and *v*

Figure 25 offers an illustration in one dimension less. We observe that events are enabled now which do not occur in any straight skeleton construction process: At the vertex *u* of ${\mathcal{R}}$ the inverse edge event ${\mathcal{E}}^{-1}_3$ takes place (in three dimensions), such that a triangular facet of ${\mathcal{Q}}^{\Delta }$ is initiated. Moreover, when ${\mathcal{R}}$ contains a local minimum (not shown in the figure) then the inverse edge event ${\mathcal{E}}^{-1}_6$ occurs, and a void in ${\mathcal{Q}}^{\Delta }$ is generated.

Note that a tetrahedron collapse ${\mathcal{E}}_6$ will take place at each local maximum of ${\mathcal{R}}$. (In particular, the polytope ${\mathcal{Q}}^{\Delta }$ vanishes in such an event when $\Delta$ achieves a global maximum.) There also are ‘switching-type’ events, which are not roof-specific but come from non-orphan-free vertex resolution, like the point-symmetric exchange of facets at the roof vertex *v* above.

The event ${\mathcal{E}}^{-1}_3$ initiates the construction of a cell in the roof complex ${\mathcal{D}}$ for ${\mathcal{R}}$, which is an orphan cell unless it merges later with a partially constructed cell bordered by ${\mathcal{Q}}$’s boundary. Likewise, ${\mathcal{E}}^{-1}_6$ and also ${\mathcal{E}}^{-1}_5$ initiate (up to four) cells in ${\mathcal{D}}$ which are possibly orphan cells. Conversely, when these three inverse events are never induced when $W^{\Delta }$ is raised, then all cells of ${\mathcal{D}}$ stay connected to the boundary of ${\mathcal{Q}}$ (but get possibly squeezed into orphan parts by switching events). More importantly, the corresponding shrinking process then always can be realized by resolving the vertices of ${\mathcal{Q}}^{\Delta }$, *without* explicit reference to ${\mathcal{R}}$. This leads to the following alternative characterization of straight skeletons in ${\mathbbm {R}}^3$.

#### Lemma 7.4

Let ${\mathcal{R}}$ be a roof for ${\mathcal{Q}}$, and let ${\mathcal{D}}$ be its roof complex. ${\mathcal{D}}$ is a straight skeleton for ${\mathcal{Q}}$ if and only if the inverse edge events ${\mathcal{E}}^{-1}_3$, ${\mathcal{E}}^{-1}_5$, and ${\mathcal{E}}^{-1}_6$ do not arise in the shrinking process defined by ${\mathcal{R}}$.

Being orphan-free is not a sufficient condition for a roof complex to be a straight skeleton, as we recall from Fig. 4(right).

### Layers in Roofs and Skeletons

For certain roof complexes, a canonical partition of the corners into so-called layers can be specified. This structural property, which we will describe now, leads to the first non-trivial upper bound on the size of straight skeletons in ${\mathbbm {R}}^3$, and also is of separate interest. For simplicity, we restrict attention to straight skeletons constructed by orphan-free vertex resolution. The results in this subsection can be extended to the class of orphan-free roof complexes.

Consider a polytope ${\mathcal{Q}}$ with connected boundary, and let ${{\mathcal {SK}}}$ be a straight skeleton for ${\mathcal{Q}}$ as above. We may assume that the cells of ${{\mathcal {SK}}}$ are topological balls: Cells are interior-connected by the orphan-free construction of ${{\mathcal {SK}}}$, and cells do not contain voids because of their monotonicity (Lemma 7.2). Moreover, tunnels in cells can be avoided by augmenting the polytope with flat edges, such that holes in facets connect to the outer facet boundary. Such edges give rise to additional sheets in ${{\mathcal {SK}}}$, but this does not alter the remaining parts of the cell complex geometrically, except that flat corners and spokes are introduced. The combinatorial complexities of ${{\mathcal {SK}}}$ and of the unrefined complex are of the same order.

There exists an *enumeration method* for the sheets in ${{\mathcal {SK}}}$, by a greedy process. As all cells in ${{\mathcal {SK}}}$ are homeomorphic to balls, we have a topological structure like for convex cells (apart from multiple adjacencies). Also, all cells are bordered by ${\mathcal{Q}}$’s boundary, which is connected by assumption. Therefore, the inner corners of ${{\mathcal {SK}}}$ can be added one by one, in a way such that each corner completes one or more sheets in ${{\mathcal {SK}}}$ adjacent to already completed ones. In particular, there exist triangular sheets incident to the boundary of ${\mathcal{Q}}$, to get the enumeration started. By Lemma 7.3, all corners of ${{\mathcal {SK}}}$ can be accessed, and hence all sheets completed, in this process.

Each corner of ${{\mathcal {SK}}}$ corresponds to the event that constructs it. At this point, we recall the notion of *contacting events* from Sect. 6.5. Global events and facet events are contacting, and all edge events are contact-free. (In a more general roof shrinking process, the events ${\mathcal{E}}^{-1}_3$, ${\mathcal{E}}^{-1}_5$, ${\mathcal{E}}^{-1}_6$, and the switching-type events are also contact-free, but they do not occur in the construction of ${{\mathcal {SK}}}$; see Lemma 7.4.) The above distinction of events into two classes induces a coloring on the inner corners of ${{\mathcal {SK}}}$. We color a corner *v* *blue* if its event is contact-free, and *red*, otherwise. The $\Delta$-value where the event happens is called the *time stamp* of *v*.

The greedy process above can be guided by corner colors. In a first round, we complete a maximal set of sheets by blue corners. We start with the triangular sheets, and collect all accessed blue corners in a set $\Gamma _1$. In a second round, we complete a set of sheets by red corners, but only while the largest time stamp in $\Gamma _1$ is not exceeded. We collect these red corners in a set $\Gamma _2$. This process is repeated by switching colors until it terminates. We claim that a unique partition of the inner corners of ${{\mathcal {SK}}}$ into subsets $\Gamma _1, \ldots , \Gamma _k$ is produced, which will be called the *layers* of ${{\mathcal {SK}}}$.

The following interpretation of layers implies the correctness of the claim. $\Gamma _1$ is a maximal set of edge events which can be carried out correctly without using any information about contacting events. $\Gamma _2$ is a neighbored set of contacting events which have been ignored meanwhile. Because we could not further enlarge $\Gamma _1$ with blue corners, $\Gamma _1$ contains corners which are local maxima in the roof for ${{\mathcal {SK}}}$, and the contacting events in $\Gamma _2$ split ${\mathcal{Q}}^{\Delta }$ (at least locally) if we execute the events in $\Gamma _1 \cup \Gamma _2$ in *shrinking order*, i.e., for ascending time stamps. This explains why we can continue with the next blue layer after collecting $\Gamma _2$, as triangular sheets with blue corners are enabled again in the complex ${{\mathcal {SK}}}\cap {\mathcal{Q}}^{\Delta }$ unless ${\mathcal{Q}}^{\Delta }$ vanishes entirely.

We obtain a linear extension of the partial order in each layer $\Gamma _i$ when sorting $\Gamma _i$ by time stamps. Concatenation of the sorted subsets now gives a unique total order of the inner corners of ${{\mathcal {SK}}}$, which we term the *greedy order* for ${{\mathcal {SK}}}$. This order will construct ${{\mathcal {SK}}}$ sheet by sheet, but differently from the shrinking order, in general.

**Fig. 26:**
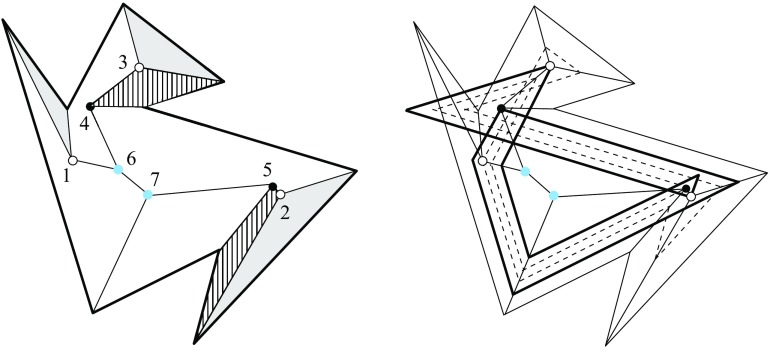
A polygon with three layers $\{1,2,3\}, \{4,5\}, \{6,7\}$ drawn as *white*, *black*, and *grey corners* (*left*). The cells completed in the first and the second layer, respectively, are *shaded* and *hatched*. On the right-hand side, the modified greedy order $1,2,3,\mathbf{5},\mathbf{4},6,7$ is simulated. The resulting polylines $\Pi (\Delta )$ and $\Pi (\Delta ')$ after the first and the second layer are shown in *bold style*. $\Pi (\Delta ')$ is a simple (actually convex) polygon, because $\{4,5\}$ is the last red layer

Visualizing the situation for straight skeletons in two dimensions (Fig. 26, left), we see edge events correspond to blue corners, and split events to red corners. All red corners are connected to the polygon boundary [4]. However, they cannot be collected in a single layer, in general, and there can be linearly many layers.

Our next aim is to realize the greedy order of events in a different geometric way, with the intention of analyzing their overall number. To this end, in the layer partition for ${{\mathcal {SK}}}$, we reverse the sorted order in each *red* layer. We then scan through this *modified greedy order* $v_1, v_2, \ldots , v_t$ of corners, and maintain a surface $\Pi (\Delta )$ in ${\mathbbm {R}}^3$ in the way described below (and illustrated in Fig. 26 for the planar case).

Initially, $\Pi (\Delta _0)$ is the boundary surface of ${\mathcal{Q}}$. For a currently processed *blue* corner $v_j$ with time stamp $\Delta _j$, we construct $\Pi (\Delta _j)$ by offsetting $\Pi (\Delta _{j-1})$ with the amount $\Delta _j - \Delta _{j-1} > 0$, and performing the edge event for $v_j$, which becomes a vertex of the surface. Self-intersections resulting from missed contacting events are *ignored*. That is, the parts of $\Pi (\Delta _{j-1})$ inverted by self-intersections extend by sliding along trisector lines, without combinatorial changes that come from contacts. $\Pi (\Delta _j)$ has planar facets, because the offset vertices for a fixed facet of ${\mathcal{Q}}$ stay in the same offset plane. Therefore, the original edge event at $v_j$ can be carried out correctly on $\Pi (\Delta _j)$. In conclusion, $\Pi (\Delta )$ is a connected, but in general self-intersecting, piecewise-linear surface during the first blue layer (and each later layer).

For each corner $v_j$ in the subsequent *red* layer, a self-intersection of $\Pi (\Delta _{j-1})$ gets untangled at $v_j$ when the surface is offset, because $\Delta _j - \Delta _{j-1}$ is now negative. The point $v_j$ becomes a vertex of $\Pi (\Delta _j)$, and a wrongly oriented triangle or tetrahedron on the surface disappears there. (Figure 15 shows two among several possible cases; an edge (left) or a triangle (right) of $\Pi (\Delta _{j-1})$ vanishes, respectively.) As before, shrinking parts—which are now inverted—and extending parts undergo edge events, without interference from contacts, and $\Pi (\Delta )$ stays connected and piecewise linear.

The philosophy behind this construction is that, because events are processed in independent portions, they can be performed consistently on $\Pi (\Delta )$ in the modified greedy order. Thereby, all events for ${\mathcal{Q}}^{\Delta }$ are eventually transformed into contact-free events for $\Pi (\Delta )$. The polytope ${\mathcal{Q}}$ has to be boundary-connected, so that the surface $\Pi (\Delta )$ can capture the mutual boundary influence in ${\mathcal{Q}}^{\Delta }$.

Note that $\Pi (\Delta )$ cannot have more edges and facets than ${\mathcal{Q}}$. No edge or facet of $\Pi (\Delta )$ ever splits, because all performed events are contact-free, and resemble the assumed orphan-free vertex resolution for ${{\mathcal {SK}}}$. In fact, the size of $\Pi (\Delta )$ decreases monotonically: Whenever a new surface edge is created, at least one edge vanishes in the same event. Concerning the size and number of layers, the following holds.

#### Theorem 7.5

Let ${\mathcal{Q}}$ be a boundary-connected polytope in ${\mathbbm {R}}^3$ with *n* facets, and let ${{\mathcal {SK}}}$ be one of its orphan-free straight skeletons. For the layers $\Gamma _1, \ldots , \Gamma _k$ of ${{\mathcal {SK}}}$ (in its tunnel-free refinement) we have $k = O(n)$ and $|\Gamma _i| = O(n^2)$.

#### Proof

${{\mathcal {SK}}}$ consists of interior-connected cells, by its orphan-free construction. We now recall that each blue layer includes corners that correspond to local maxima in the skeleton roof for ${{\mathcal {SK}}}$. Therefore, at least one cell of ${{\mathcal {SK}}}$ gets fully completed, if the events in this layer were performed in shrinking order. In the last blue layer, at least 4 cells get completed when the offset polytope vanishes entirely. The number of cells of ${{\mathcal {SK}}}$ is at most *n*, and we obtain $k \le 2(n-3)$.

For the cardinalities of layers we give the following arguments. While staying within a fixed layer $\Gamma$, we offset $\Pi (\Delta )$ in a fixed direction. Assume first that $\Gamma$ is a blue layer. For each corner *v* in $\Gamma$, an edge event takes place, and at least one edge *e* on $\Pi (\Delta )$ vanishes in this event. Consider the bisector plane $B_{ij}$ that contains the edge *e*. We study the interplay on $B_{ij}$ while $\Delta$ increases. Let $e \subset \ell = H_i^{\Delta } \cap H_j^{\Delta }$, and let $H_k^{\Delta }$ and $H_m^{\Delta }$ be the two offset planes that define the endpoints of *e*. The three lines $\ell$ and $\ell ' = H_k^{\Delta } \cap B_{ij}$ and $\ell '' = H_m^{\Delta } \cap B_{ij}$ arrive at *v* at the same time, causing *e* to vanish. Edge *e* may reappear immediately, for example when *v* is a saddle vertex, but this can happen only once. Otherwise, as no contacting events can hinder their influence, at least one of $\ell '$ and $\ell ''$ will reach any further point $x \in B_{ij}$ earlier than $\ell$. This implies that $B_{ij}$ contributes at most two edges to $\Pi (\Delta )$ while $\Gamma$ is processed. Hence at most two corners in $\Gamma$ can be charged to $B_{ij}$. From $i < j \le n$ we conclude that the cardinality of a blue layer does not exceed $2 \cdot {n \atopwithdelims ()2}$.

If $\Gamma$ is a red layer, then its number of corners is bounded from above by the size of the pattern of self-intersection on $\Pi (\Delta )$. This size is $O(n^2)$, because $\Pi (\Delta )$ has only *O*(*n*) edges and facets. $\square$

From Theorem 7.5 an upper bound of $O(n^3)$ on the size of (orphan-free) straight skeletons can be inferred. We have stated this result in Theorem 5.3 in Sect. 5.

The number of layers depends on the shape of the polytope ${\mathcal{Q}}$. Let us call ${\mathcal{Q}}$ *near-convex* if it allows for some straight skeleton with a single (blue) layer. That is, no contacting events occur, hence the greedy order is identical to the shrinking order. For example, convex polytopes are near-convex. Also, the surface $\Pi (\Delta )$ is intersection-free once the last red layer has been processed, and $\Pi (\Delta )$ then bounds a near-convex polytope; see Fig. 26 again. Recognizing and characterizing near-convex polytopes, or polygons, are interesting problems; an application is described in Sect. 10.2. From Theorem 7.5 we obtain the following tight bound.

#### Corollary 7.6

If ${\mathcal{Q}}$ is a near-convex polytope then (at least) one of its straight skeletons can be constructed with $O(n^2)$ edge events. No facet events or global events are involved.

For general roof complexes for ${\mathcal{Q}}$, the layer partition needs not exist: The shrinking process can introduce voids in ${\mathcal{Q}}^{\Delta }$, and cells are not always bordered by ${\mathcal{Q}}$’s boundary. In case of its existence, the partition may consist of a super-linear number of layers, if the complex contains a large number of orphan cells. Likewise, when vertex resolution is not orphan-free in the straight skeleton construction, then cells get locally but not necessarily globally completed in blue layers. Examples of size $\Theta (n^3)$ exist in these cases; see Sect. 8.

### Computing Roof Complexes

From the algorithmic point of view, roof complexes provide an alternative way of computing monotone decompositions of a polytope ${\mathcal{Q}}$, without resorting to straight skeleton algorithms.

The surface of a roof for ${\mathcal{Q}}$ consists of (the union of) faces from the arrangement, ${\mathcal{L}}({\mathcal{Q}})$, of the *n* roof hyperplanes $L_1, \ldots , L_n$ in ${\mathbbm {R}}^4$; see Sect. 7.1. As a consequence, the combinatorial size of any roof complex is bounded from above by the combinatorial size of ${\mathcal{L}}({\mathcal{Q}})$, which is $\Theta (n^4)$.

To construct a roof for ${\mathcal{Q}}$, one can compute the arrangement ${\mathcal{L}}({\mathcal{Q}})$ in $O(n^4)$ time in a preprocessing step [20], and then apply the cell adding technique from Sect. 3.2 directly (rather than using it in one dimension lower for vertex resolution). We start with the *zone* in ${\mathcal{L}}({\mathcal{Q}})$ for the horizontal hyperplane $W^0$, that is, the collection ${\mathcal{K}}$ of arrangement cells intersected by $W^0$. Let $\overline{{\mathcal{K}}}$ be the union of the cells in ${\mathcal{K}}$. As long as $\overline{{\mathcal{K}}}$ does not define a valid function on ${\mathcal{Q}}$, we continue adding to ${\mathcal{K}}$ cells of ${\mathcal{L}}({\mathcal{Q}})$ incident to (three-dimensional) facets which violate this property. This process terminates with a collection of cells whose boundary surface defines a roof for ${\mathcal{Q}}$.

Depending on the kind of cell complex we are aiming at, we can continue the process. For simplicity, let us assume that ${\mathcal{L}}({\mathcal{Q}})$ is generic. As long as roof vertices of degree larger that 4 exist, we can add respective cells, at the same time restoring with additional cells (if necessary) the property that $\overline{{\mathcal{K}}}$ defines a function. This yields a roof complex ${\mathcal{D}}$ with inner corner degree 4.

Still, the complex ${\mathcal{D}}$ may contain orphan cells, which can be repaired by adding cells incident to roof facets which do not intersect $W^0$. An orphan-free complex ${\mathcal{D}}^*$ with at most *n* cells is obtained, where the requirement that $\overline{{\mathcal{K}}}$ gives a valid roof is then automatically fulfilled.

#### Lemma 7.7

Let ${\mathcal{Q}}$ be an (arbitrary) polytope with *n* facets. A decomposition ${\mathcal{D}}^*$ of ${\mathcal{Q}}$ into *n* monotone polytopal cells and inner corner degree 4 can be computed in $O(n^4)$ time.

The cell complex ${\mathcal{D}}^*$ is not unique, but rather depends on the order in which cells are added. To recognize whether ${\mathcal{D}}^*$ is a straight skeleton for ${\mathcal{Q}}$, we can inspect the inner corners of ${\mathcal{D}}^*$ concerning the event they embody. By Lemma 7.4, the answer is affirmative if and only if the events ${\mathcal{E}}^{-1}_3$, ${\mathcal{E}}^{-1}_5$, and ${\mathcal{E}}^{-1}_6$ are not encountered.

It remains unclear how to specify an order on the cells of ${\mathcal{L}}({\mathcal{Q}})$ that produces a straight skeleton. Counterexamples exist, already in two dimensions [4], to the conjectures that the *minimal* or the *maximal* orphan-free roof (seen as functions on ${\mathcal{Q}}$) yield a straight skeleton. The (overall) minimal roof ${\mathcal{R}}^-$, or maximal roof ${\mathcal{R}}^+$ respectively, can contain orphan facets and thus seem less relevant in practical applications.

The following observations indicate that it may be hard to improve the upper size bound of $O(n^4)$ for general roof complexes. By a result in [11], the combinatorial size of such a complex in ${\mathbbm {R}}^1$ (which is a partition of a line segment) can already be near-quadratic in *n*. Also, an attempt to apply the *lower envelope* bound for *n* linear partial functions in ${\mathbbm {R}}^4$, which is $O(n^{3+\varepsilon })$ [28], is doomed to fail when only ${\mathcal{Q}}$ is given, without additional knowledge about the roof complex. This is already true in two dimensions [4]. Finally, the large size of *k*-levels in hyperplane arrangements in ${\mathbbm {R}}^4$ is discouraging. (The *k*-level is composed of all arrangement faces which lie below exactly $k-1$ hyperplanes, and the best known upper bound on its complexity is $O(n^2 k^{2-\varepsilon })$; see [1].) On the other hand, a roof can never be a subset of any *k*-level for fixed *k* when ${\mathcal{Q}}$ is nonconvex: At each locally nonconvex part of ${\mathcal{Q}}$, some of the hyperplanes $L_i$ will transversely cut the roof surface, and the intersected roof faces then belong to different levels. When ${\mathcal{Q}}$ is a *convex* polytope then the $(n-1)$-level of ${\mathcal{L}}({\mathcal{Q}})$, which is the lower envelope of $L_1, \ldots , L_n$, constitutes the only existing roof.

The cell adding method is conceptually simple and flexible, and may construct polytope decompositions of small size in many cases. For example, the size of orphan-free 3D straight skeletons is bounded by $O(n^3)$ (Theorem 5.3), and tends to be subquadratic in most examples (Sect. 10.1). This raises the question of whether a possible *tracing algorithm*, based on the enumeration method for sheets in Sect. 7.2, can avoid the $\Theta (n^4)$ time (and space) barrier in Lemma 7.7, and achieve an output-sensitive runtime. The layer partition of corners for orphan-free roof complexes even shows that their computation can in principle be parallelized; each layer is structured only by a partial order, rather than by a total order. Unfortunately, it seems that finding the layer partition requires prior knowledge of the roof complex.

**Fig. 27:**
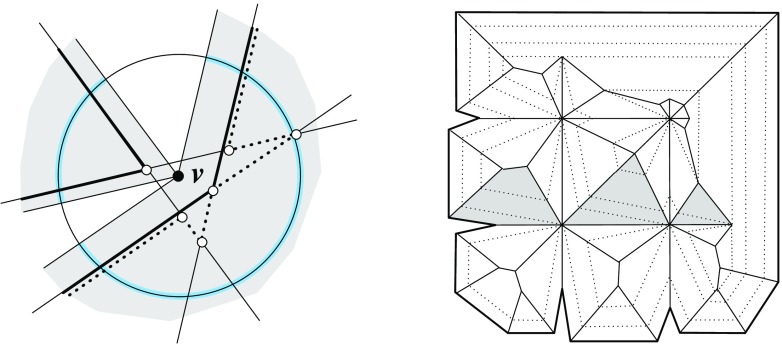
A multiple split event for a weighted polygon (*left*). The vertex *v* resolves into up to 6 vertices marked with $\circ$. The *right-hand side* shows a straight skeleton that contains a quadratic number of orphan cells, because multiple split events are not resolved in an orphan-free way

## Weighted Setting

A concept already considered in [3, 21] is to individually tune the speeds of the edges of a polygon in its shrinking process. Naturally, doing the same for the facets of a polytope raises our interest. In practical applications, such *weighted* mitered offset surfaces might be of particular interest, as they offer additional flexibility for adapting to given needs.

### (Weighted) Polygons Revisited

As a warm-up, we reconsider the polygon case in the light of Definition 3.1. Let a *polygon*, ${\mathcal{P}}$, be the two-dimensional equivalent of a polytope as defined in Sect. 2. ${\mathcal{P}}$ may contain holes, and also touching vertices which then belong to more than two edges. We equip each edge $e_i$ of ${\mathcal{P}}$ with an individual weight $w_i > 0$, which represents its velocity in the shrinking process. Polygon vertices move on weighted angle bisectors, which are straight lines such that the weighted straight skeleton is a piecewise linear structure; see also Sect. 4.1.

For a given vertex *v* of ${\mathcal{P}}$, its *valid offsets* are now given by the (dimension-independent) conditions in Definition 3.1. Let ${\mathcal{A}}(v)$ be the respective arrangement of offset lines. Like in the unweighted case, ${\mathcal{A}}(v)$ is combinatorially independent from the offset parameter $\Delta$. Note that ${\mathcal{A}}(v)$ may contain parallel (non-identical) lines $h^{\Delta }_i$ and $h^{\Delta }_j$ now, which are at distances $w_i \ne w_j$ from *v*. The spherical polygon becomes a possibly disconnected circular domain; see Figs. 27(left) and 28(left).

If no degeneracies occur in the shrinking process for ${\mathcal{P}}$, then vertex offsets are always unique: ${\mathcal{A}}(v)$ is defined by only two lines for initial events, and by three lines for non-initial events. In the latter case, ${\mathcal{A}}(v)$ contains a single bounded (triangular) cell *t*. It is easy to verify that *t* either must, or cannot, contribute to an inner offset which is radially visible. That is, edge events and split events are unique.

For *multiple events*, Definition 3.1 offers more generality than the standard polygon offsetting process. There is ambiguity caused by orphan edges, also for unweighted polygons. As an example, see Fig. 27(left) for a multiple split event, which is also called a *vertex event* in the literature [21]. Edge events can occur multiply as well, when two or more edges of ${\mathcal{P}}$ vanish at the same time. Such an event can be resolved either as in Fig. 5(right), or as in Fig. 25(middle) where orphan cells of the skeleton are created. The latter instance might be called a *switching event*, as a point-symmetric exchange of edges takes place. Switching events have the advantage that they keep the straight skeleton of ${\mathcal{P}}$ connected when parallel edges get merged, because no vertex of the offset polygon flattens out. Otherwise, the skeleton disconnects into a forest, even in the unweighted case.

On the other hand, skeleton cells do not stay *interior-connected* when orphan edges are allowed in events (but remain connected via the resolved vertex). In fact, a fixed edge $e_i$ of ${\mathcal{P}}$ can trace out $\Omega (n)$ concatenated orphan cells when ${\mathcal{P}}$ has *n* edges; see Fig. 27(right). Multiple events can lead to planar straight skeletons whose combinatorial complexity is $\Theta (n^2)$.

**Fig. 28:**
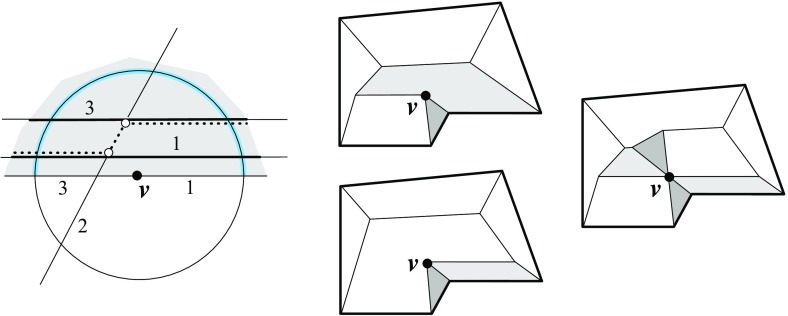
The situation for parallel polygon edges with weights 3 and 1. There are three valid offsets for the sliding event at vertex *v* (*left*). Two of them are the *horizontal lines* shown in *bold*. The third offset is drawn with *dotted lines*, and leads to a weighted skeleton with orphan cells (*right*)

In the weighted case, ambiguity arises in a generic way in a new edge event: The offsets of two parallel edges $e_i$ and $e_j$ of ${\mathcal{P}}$ with $w_i \ne w_j$ can merge into one edge, because the edge that separated them vanishes at vertex *v*. See Fig. 28 for this *sliding event*, its three radially visible offsets, and the different skeletons generated by them. The sliding event does not disconnect the weighted straight skeleton. In the offset line arrangement ${\mathcal{A}}(v)$, the triangular cell *t* degenerates to two slabs separated by the third offset line. Therefore, this type of ambiguity cannot be fixed by ruling out orphan edges owing to the more general definition of an offset boundary; the arity of the sliding event is 2.

### Weighted Skeletons in 3-Space

We now study the effect of weighting on a polytope ${\mathcal{Q}}$ in ${\mathbbm {R}}^3$. The discussion in the preceding subsection already indicates that there are no substantial differences to the unweighted case.

Most importantly, Definition 3.1 extends to weighted offset surfaces in ${\mathbbm {R}}^3$, also in the case where the offset arrangement ${\mathcal{A}}(v)$ contains parallel planes that stem from differently weighted parallel facets of ${\mathcal{Q}}$. Moreover, the existence is guaranteed: The proof of Theorem 3.3 generalizes straightforwardly, which also implies that weighted offset surfaces have to exist in orphan-free form. Weighted straight skeletons for ${\mathcal{Q}}$ therefore are well-defined cell complexes, for arbitrary positive facets weights.

The generic condition (Definition 6.1) now has to involve both weights and offset plane positions. In particular, two parallel offset planes $H^{\Delta }_i$ and $H^{\Delta }_j$ with the *same* orientation may identify for some value $\Delta > 0$ if $w_i \ne w_j$, and are counted as two different planes. Note that a non-generic weighting can force three offset planes to intersect in a common line for all values of $\Delta$.

Weighting legitimates more offset surfaces, because for general weights new combinatorics for a polytope vertex can arise. This is also true for orphan-free vertex resolution, which will be assumed in the sequel. For example, we can now obtain the two surfaces in Fig. 6(middle), by assigning a weight $w > 1$ to the facet *f* of the degree-5 vertex *v*, and weight 1 to its four other facets. Stated in different terms, for such a weighting the arity of the initial event at *v* is upgraded from 1 to 2. The spherical polygon for *v* stays the same, of course, but its bisector graphs change as being defined by *weighted* angle bisector planes. The vertex splitter in Sect. 4.2 will automatically recognize this situation, by checking the validity of candidate graphs with respect to the altered spherical embedding; cf. Lemma 4.5.

As in two dimensions, new weighting-specific events related to parallelism arise in ${\mathbbm {R}}^3$. For example, when we resolve the apex of a pyramid based on the polygon ${\mathcal{P}}$ in Fig. 28(right) in an initial event, we obtain the three offset surfaces shown, for a fixed suitable set of facet weights. This is not possible when all weights have to be the same, as we recall from Sect. 4.1.

Similarly, when the polygon ${\mathcal{P}}$ is a facet (or a hole in a facet) of the offset polytope ${\mathcal{Q}}^{\Delta }$, then facet weights can be adjusted to let such a *sliding edge event* happen at vertex *v*, as a non-initial event. Thereby, the two parallel edges of ${\mathcal{P}}$ arrive at *v*, and in the orphan-free version they merge into a single edge. This edge can take over the offset movement of either merged part, which results in two different offset polytopes. In the weighted straight skeleton, three spokes are completed at *v*, and one new spoke (among two possible collinear choices) is constructed *in one go* at time $\Delta$. Vertex *v* becomes a skeleton corner of degree 4, like for unweighted skeletons. The spoke graph does not continue at *v*. This event is generic, belongs to the class of edge events because one edge vanishes, and is of arity 2. Notice that ${\mathcal{A}}(v)$ contains no parallel planes in this case, but three planes parallel to the same line.

There also exists a non-initial generic *sliding facet event*. It stems from two parallel planes in ${\mathcal{A}}(v)$, and is the 3D analogue of the planar sliding event. This event resembles the facet/facet collision event in Sect. 6.4. However, the identifying offset planes have the same orientation now, such that the event is not global. It can be categorized as a facet event, because two polytope facets merge into a single facet of ${\mathcal{Q}}^{\Delta }$. On the other hand, there is similarity to a simultaneous edge event, because several polytope edges incident to the merging facets vanish at different vertices of ${\mathcal{Q}}^{\Delta }$. The event is binary, because the new facet might proceed with either speed of its merged parts. For each vanished edge, a skeleton corner is generated where 4 spokes are completed, such that the spoke graph construction does not continue at these corners (like in the events ${\mathcal{E}}_5$, ${\mathcal{E}}_6$, and ${\mathcal{G}}^{-1}_{\textsc {k}}$).

We recall from Sect. 6.3 that certain facet events, or their inverses, also merge two parallel facets into one. If these facets carry different weights, then the event is not generic any more, though the structure of the (now binary) event does not change. By weighting other facets, however, a combinatorially different bisector graph can be obtained for some facets events, which then stay generic and unary. For instance, this happens for the inverse of the facet event ${\mathcal{F}}_{\textsc {v}}$ (but in its *non-complementary* setting).

No other new weighted events exist in the generic case. ${\mathcal{A}}(v)$ cannot contain 3 or 4 parallel planes, and 4 nonparallel planes which are parallel to the same line lead to the global event of type collinear-edge/edge collision, in Sect. 6.4. Edge events and global events where ${\mathcal{A}}(v)$ lacks all kinds of parallelism remain combinatorially unaffected when weighted, and their arity is preserved. Global events with geometric degeneracies do not change either.

In summary, the *initial* events can change when weighted, but generic *non-initial* events do not, except possibly for facet events, albeit new event types are enabled. The incidence structure of the produced weighted straight skeleton, ${{\mathcal {SK}}}_w$, of ${\mathcal{Q}}$ is that of a cell complex as in Sect. 5.2. Generally, the properties of ${{\mathcal {SK}}}_w$ are almost the same as of an unweighted skeleton. All cells of ${{\mathcal {SK}}}_w$ are bordered by ${\mathcal{Q}}$’s boundary, because weighted offset planes still contribute continuously during the construction of ${{\mathcal {SK}}}_w$ in the shrinking process. This property is important. It implies that there are at most *n* interior-connected cells, when the construction obeys orphan-free vertex resolution. Also, the property is needed for Lemma 5.2 to hold: The spoke graph of ${{\mathcal {SK}}}_w$ is connected to ${\mathcal{Q}}$’s boundary. In fact, the layer construction in Sect. 7.2 extends (see also below), such that the $O(n^3)$ upper bound in Theorem 5.3 is valid for the combinatorial complexity of weighted straight skeletons in ${\mathbbm {R}}^3$.

When orphan facets may be produced in the resolution of vertices, then a cubic lower bound exists. The construction in Fig. 27(right) can be generalized to ${\mathbbm {R}}^3$ in an obvious way, which results in a straight skeleton of size $\Theta (n^3)$ even when all weights are put to 1.

The only larger difference to the unweighted case is the lack of monotonicity of the skeleton cells (Lemma 7.2). For general facet weights for ${\mathcal{Q}}$, only the cell of the facet with smallest weight, which corresponds to the steepest roof hyperplane, is monotone. In particular, this implies that the cells in ${{\mathcal {SK}}}_w$ can have *voids*—but only if ${\mathcal{Q}}$ does the same, similar to the two-dimensional case. Hence Lemma 7.3 still extends to the spoke graph of weighted roof complexes *without* orphan cells.

No other proofs in earlier sections are based on the monotonicity property, such that the respective results carry over to the weighted setting. For instance, if ${\mathcal{Q}}$ is a boundary-connected polytope, then none of its offset polytopes contains voids (Lemma 6.5), so that all cells in ${{\mathcal {SK}}}_w$ are still void-free. (This is needed in the derivation of the cubic size bound in Theorem 5.3.) Also, the computation of roof complexes extends (Lemma 7.7), and works in $O(n^4)$ time for weighted roofs.

In this context, let us mention an optimality property of weighted roofs for *convex* polytopes ${\mathcal{Q}}$, proved in [7]. Let ${\mathcal{Q}}$ have *n* facets and volume 1. For any positive numbers $\lambda _1, \ldots , \lambda _n$ with $\sum \lambda _i = 1$, there exists a weighted roof complex for ${\mathcal{Q}}$ such that its cells $C_i$ have volumes $\lambda _i$. More precisely, facet weights $w_1, \ldots , w_n$ for ${\mathcal{Q}}$ can be found, such that the resulting weighted roof complex for ${\mathcal{Q}}$ (which is now unique and identical to ${{\mathcal {SK}}}_w$) decomposes ${\mathcal{Q}}$ into prescribed shares. This result does not generalize to nonconvex polytopes, because ${{\mathcal {SK}}}_w$ then can change discontinuously with the weighting.

## Higher Dimensions

The concepts of polytope, radial visibility, and arrangement of planes generalize to Euclidean *d*-space ${\mathbbm {R}}^d$, for $d \ge 4$. As a consequence, many of the results in this paper are inherently dimension-independent, and have analogues in higher dimensions. This section comments on some implications.

The proof of the existence of mitered offset surfaces for a polytope vertex *v*, being based on its offset arrangement ${\mathcal{A}}(v)$, generalizes directly to arbitrary dimensions *d*. This also includes the orphan-free case, and positively weighted surfaces. Straight skeletons of nonconvex polytopes in ${\mathbbm {R}}^d$ therefore are well-defined geometric structures, both in their weighted and unweighted form. In a suitably generalized generic case (which we will assume below), straight skeletons are simple cell complexes, with exactly ${{d+1} \atopwithdelims ()j}$ skeleton faces of dimension *j* incident to each inner corner *v*. In particular, exactly $d+1$ cells and spokes, respectively, meet at *v*. Geometric—but no combinatorial—degeneracies can occur in the cell structure, due to doubly-adjacent faces of various dimensions; cf. Sect. 5.2.

The regular structure of a straight skeleton stands in contrast to the incidence structure of the medial axis of ${\mathcal{Q}}$, and the high algebraic degree of its components in ${\mathbbm {R}}^d$, unless the defining Euclidean metric for the medial axis is replaced by some piecewise linear metric [2]. Note that a polytope ${\mathcal{Q}}$ in ${\mathbbm {R}}^d$ with *n* facets can have $\Omega (n^{\lfloor \frac{d}{2} \rfloor })$ vertices. On the other hand, the number of cells in a straight skeleton (and also in the medial axis) of ${\mathcal{Q}}$ can be at most *n*, and the number of skeleton corners is trivially bounded by ${n \atopwithdelims (){d+1}}$.

The structure of an event at a vertex *v* of ${\mathcal{Q}}$ in its shrinking process is determined by the arrangement ${\mathcal{A}}(v)$. For non-initial events, ${\mathcal{A}}(v)$ contains $d+1$ hyperplanes which define a single *d*-simplex as the only bounded cell. This implies that, interestingly, such events are either unary or binary; there exist at most two resolution surfaces for *v*, like in the three-dimensional case (cf. the proof of Lemma 6.3). Initial events, of course, can be arbitrarily complex and have a high arity.

Our criteria for event classification are still meaningful. Concerning contacting events, we have global events where combinatorially unrelated parts of the offset polytope ${\mathcal{Q}}^{\Delta }$ collide, and facet events where the same happens within some facet of ${\mathcal{Q}}^{\Delta }$. Edge events constitute the remaining class, where necessarily some edges of ${\mathcal{Q}}^{\Delta }$ have to vanish, and where events are contact-free. Inverse events are well-defined via outer offsets or, equivalently, by generalizing Definition 6.2. The class of facet events may be further refined, by individually referring to face dimensions *j*, for $2 \le j \le d-1$.

Clearly, the number of event types within each class increases with the dimension. There are ${{d+1} \atopwithdelims ()2}$ potential edge events, according to the same number of edges of a *d*-simplex. Also, there are (at least) quadratically many facet events and global events, because contacting faces of different dimensions have to be paired up. This is probably also the most suitable method for detecting these events, as higher-dimensional data structures tend to lose their efficiency in practice. However, all edge events (and all events from other classes where some edges vanish) can still be detected directly.

As bisector graphs are not appropriate any more, the algorithm of choice for event processing is the cell adding procedure using the arrangement ${\mathcal{A}}(v)$; see Sects. 3.2 and 4.2. For non-initial events, ${\mathcal{A}}(v)$ contains exactly $2^{d+1}-1$ cells, such that the (at most 2) valid offset surfaces of a vertex *v* can be found reasonably quickly for small dimension *d*. Also for initial events, where $O(m^d)$ cells have to be added, this method is still plausible when polytope vertices are of constant degree $m \ge d$. This particularly concerns polytopes in ${\mathbbm {R}}^4$, which might be the most interesting case. As with the bisector-graph based algorithm in Sect. 4.2, the type of an event needs not be known in advance, which makes a uniform treatment of events possible.

The concept of roofs and their complexes inside a polytope ${\mathcal{Q}}$ generalizes, and defines a decomposition of ${\mathcal{Q}}$ into polyhedral cells, which are monotone in the unweighted case. Simple cell complexes as in Lemma 7.7 can be computed in $O(n^{d+1})$ time, once more by applying the cell adding technique, but now to the arrangement ${\mathcal{L}}({\mathcal{Q}})$ of the *n* roof hyperplanes in ${\mathbbm {R}}^{d+1}$. Again, the main part of the algorithm is the construction of a hyperplane arrangement.

Finally, the sheet enumeration method from Sect. 7.2 extends to the 2-faces in straight skeletons of any dimension (including the planar case). Skeleton cells can be forced to be topological balls, by the augmentation of polytope facets with flat faces. Then blue triangles generated by edge events always have to be present in an orphan-free skeleton, and $(j+1)$-sheets can be constructed by completing *j*-sheets, for $j \ge 2$. That is, the layer partition of corners and their greedy order exist. Simulating the greedy order by maintaining a surface $\Pi (\Delta )$ in ${\mathbbm {R}}^d$, we see that the number of layers stays linear in *n* for (weighted) straight skeletons, because each blue/red pair of layers can be charged to the construction of some skeleton cell again. The count for corners per blue layer can be done with respect to the angle $(d-1)$-sector planes of the polytope ${\mathcal{Q}}$ (for example, the ${n \atopwithdelims ()3}$ trisector planes in ${\mathbbm {R}}^4$). The same arguments as in the proof of Theorem 7.5 apply, and lead to an upper bound of $O(n^{d-1})$ for a fixed layer. We obtain the following general theorem for the size of straight skeletons.

### Theorem 9.1

Let ${\mathcal{Q}}$ be a boundary-connected polytope in ${\mathbbm {R}}^d$ with *n* facets, for arbitrary constant dimension *d*. Every (weighted or unweighted) straight skeleton for ${\mathcal{Q}}$ is of combinatorial complexity $O(n^d)$ in the orphan-free setting.

Roof surfaces can be viewed as *monotone terrains* in (non-vertical) hyperplane arrangements in ${\mathbbm {R}}^{d+1}$, which are generalizations of *monotone paths* in line arrangements [11]. They range between zones and levels; see Sect. 7.3. As mentioned there, monotone paths in ${\mathbbm {R}}^2$ can be long, and the size of a *k*-level in an arrangement of planes in ${\mathbbm {R}}^3$ can be super-quadratic. But when only *unbounded* terrain facets are present, then obviously the terrain size in ${\mathbbm {R}}^2$ is 2, and *O*(*n*) in ${\mathbbm {R}}^3$ because the edges of the terrain form a planar graph with at most *n* faces. It remains unclear what happens in higher dimensions. However, from the straight skeleton bound in Theorem 9.1 we conjecture that monotone terrains composed of *n* unbounded facets behave like zones, having a combinatorial complexity of $O(n^d)$ in ${\mathbbm {R}}^{d+1}$.

## Practical Issues

In this section, we report on some experimental results and potential applications. Many more details concerning implementation aspects and experiments are provided in [33].

### Implementation

We have implemented an algorithm that computes (weighted) straight skeletons in ${\mathbbm {R}}^3$. To be able to accept input polytopes with vertices of higher degree, the algorithm also works in the non-generic case. This is eased by the fact that once an event has been detected, its processing needs not be adapted to its anatomy; all events are treated uniformly with the vertex splitter in Sect. 4.2. Constant time per event suffices, when the degree of the resolved vertex is a constant.

The detection of events, on the other hand, is a notoriously difficult and time-consuming task, as is well known from the planar case. We have mentioned some possibilities for speed-up along with the event description. The problem is in finding the contacting events, especially the global ones. It seems hard to capture the interplay of the reflex faces of the polytope ${\mathcal{Q}}$ with a structure like the motorcycle graph [21, 32], which reduces the worst-case runtime in two dimensions. In practice, a suitable volume tetrahedrization of ${\mathcal{Q}}$ should give a satisfactory detection method, with output-sensitive behavior and sublinear average runtime per event for most boundary-meshed polytopes. We have only implemented the straightforward method, as our main interest here is in the structural and quantitative properties of the computed skeletons. For facet events the detection is still fast, because facet sizes can be considered a constant for most polytope data.

**Fig. 29:**
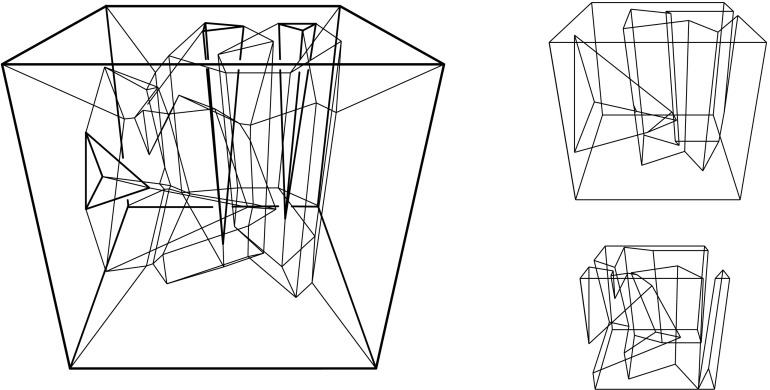
The polytope ‘Spiky’ with its unweighted straight skeleton (*left*), and the two offset polytopes after 7 and 16 events, respectively (*right*)

Figure 29 displays an example of the output. (In fact, most illustrations in Sect. 6 have been produced automatically.) The polytope ${\mathcal{Q}}$ is a cube with three pyramidal pits. It has 20 vertices which all have degree 3. Therefore, no initial events need to be performed, and the straight skeleton is constructed with a total of 49 non-initial events. The global events include 3 piercing events and 1 kissing event, the latter leading to a merge of two tunnels. There are 22 facet events, where 11 split the offset polytope. The edge events list as 10 of type ${\mathcal{E}}_3$, 8 of type ${\mathcal{E}}_6$, and only 5 where a single edge vanishes. Comparing the number of splits to the number of tetrahedra collapses, we see that $1+11-8 = 4$ splits must be local and destroy tunnels in the offset polytope. There are more contacting events than edge events, due to the extreme shape of ${\mathcal{Q}}$. The number of skeleton corners is vertices plus non-initial events, which gives 69. The skeleton is unique in this case, because there are no initial events, and the binary saddle vertex event ${\mathcal{E}}_{1b}$ does not occur. Observe that two facets of ${\mathcal{Q}}$ have holes (the topmost facet, and the one on the left side), and their cells contain tunnels. Indeed, there is no greedy order of the events; its existence has to be restored by adding three flat edges on the boundary of ${\mathcal{Q}}$.

**Fig. 30:**
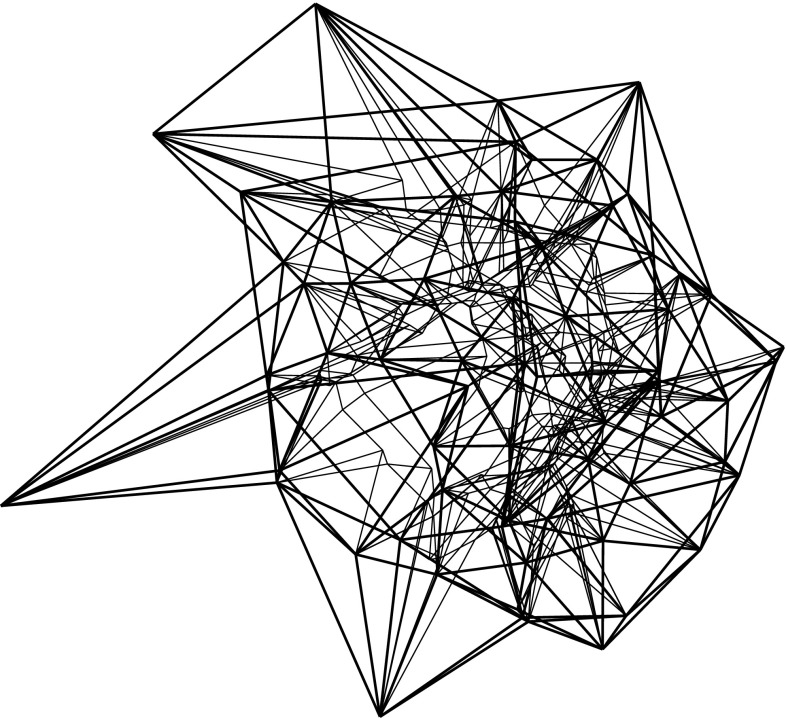
The polytope ‘Verwortakelt’ has 66 vertices and a triangulated boundary. In the construction of its straight skeletons, the number of edge events always by far dominates the number of facet events and global events

**Fig. 31:**
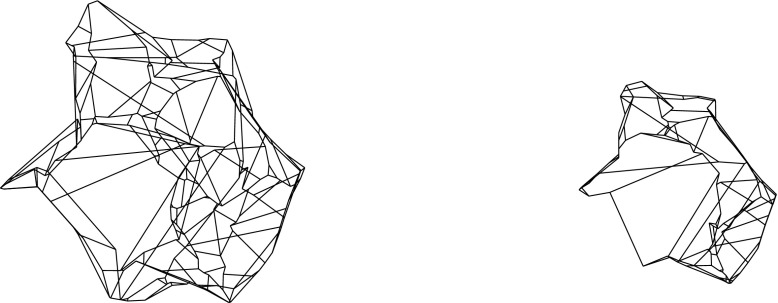
As contacting events with topological changes occur rarely for the polytope in Fig. 30, its offset polytopes keep the approximate shape for a long time

We have tested various polytopes of different sizes, mostly boundary-triangulated and modeling common shapes, but also polytopes of extreme shape, like the ones in Fig. 10 (ID: *Pizza Box*  in the table below) and Fig. 29 (ID: *Spiky*), and related ones. One of our larger boundary-triangulated input polytopes (ID: *Verwortakelt*) is displayed in Figs. 30 and 31. Different straight skeletons for each polytope have been computed, including weighted skeletons, and their corners/spokes/sheets counted, along with the number *L* of layers if they exist. Except for extreme polytopes, the number of corners always was a small multiple (less that 10) of the number of polytope vertices. The (maximal) runtime is manageable for moderate polytope sizes. A brief excerpt of our experiments is given in the following table.

**Table:** 

ID/Vertices	SK convex	SK reflex	SK weighted	*L*	sec
*Spiky*/**20**	**69**/108/70	**69**/108/70	**42**/54/46	–	0.5
*Convex*/**24**	**60**/84/61	**60**/84/61	**44**/52/45	1	0.1
*Pizza Box*/**32**	**135**/222/136	**135**/222/136	**51**/54/55	10	3.5
*Pizza Cube*/**44**	**161**/256/162	**161**/256/162	**152**/238/153	19	4.1
*Asteroid*/**66**	**373**/740/562	**443**/880/635	**375**/742/563	6	22.7
*Verwortakelt*/**66**	**464**/922/661	**540**/1074/749	**468**/930/661	9	50.5
*Lion*/**89**	**501**/996/760	**566**/1126/830	**498**/990/759	7	46.3
*Creature*/**99**	**516**/1026/810	**596**/1186/894	**518**/1030/812	6	47.4
*Statue*/**142**	**946**/1886/1372	**1087**/2168/1517	**941**/1876/1366	–	184.8
*Bunny*/**152**	**919**/1832/1371	**961**/1916/1416	**931**/1856/1383	–	162.7

Numbers of vertices and corners are indicated in bold

Events with arity larger than 1 give choice among different (orphan-free) offset surfaces, especially for initial events. For example, we can maximize the number of *convex edges*, or *reflex edges* respectively, at each event. As can be seen from the table (Columns 2 and 3), the former strategy led to fewer events, and thus to straight skeletons of smaller combinatorial size, apart from the first four polytopes whose skeletons are unique. This choice also tends to keep the *volume* large, whereas the polytope is ‘slimmed down’ more quickly when reflex edges are maximized. However, extreme volumes are not achieved; there exist counterexamples for initial events.

Of course, the volume can be maximized (or minimized) directly in each event. Unfortunately, this local optimization strategy does not guarantee that the volume of the offset polytope ${\mathcal{Q}}^{\Delta }$ is kept maximal (minimal) for *all* values of $\Delta$. Similarly, choosing fewer convex edges in some event can lead to later events which generate a polytope with more convex edges. Concerning skeleton size, extreme volume gave quite similar counts as extreme edge convexity, but none of these criteria produced the smallest (respectively largest) skeleton in all examples.

The effect of weighting on the straight skeleton can be seen in Column 4. In the respective polytope, all facets carry unit weight but one, whose weight was put to 10. This can lead to a drastic reduction of the skeleton size (Rows 1 to 3), when the heavy-weighted facet defines a large cell and extrudes many other facets which had larger cells in the unweighted skeleton. The effect gets slightly stronger when convex offset edges are maximized, wherefore we used this construction rule.

The layer count *L* in Column 5 shows that usually only a few layers are present, unless the polytope has an extreme shape. Convex polytopes, like the one in Row 2, have a single layer; see Corollary 7.6. For three test polytopes the greedy order does not exist, because tunnels in skeleton cells arise. The number of layers tends to be larger when reflex offset edges are maximized; we used this choice in the table.

Concerning optimization criteria, let us mention a relation between the volume of the offset polytope ${\mathcal{Q}}^{\Delta }$ and its *surface area*. Denote with $\Sigma$ the union of all offset facets created in ${\mathcal{Q}}^{\Delta +t}$ when a vertex *v* of ${\mathcal{Q}}^{\Delta }$ is resolved. As $\Sigma$ is radially visible, the volume $V(\Sigma )$ of the pyramid with base $\Sigma$ and apex *v* is the sum of the volumes of the respective pyramids based on $\Sigma$’s facets. That is, $V(\Sigma ) = A(\Sigma ) \cdot \frac{t}{3}$, when $A(\Sigma )$ is the area of $\Sigma$. Considering two offset surfaces $\Sigma _1$ and $\Sigma _2$ for *v*, the (local) volume difference of the offset polytopes ${\mathcal{Q}}_1^{\Delta }$ and ${\mathcal{Q}}_2^{\Delta }$ therefore is $-(A(\Sigma _1)-A(\Sigma _2)) \cdot \frac{t}{3}$. This implies that maximizing the volume of an offset polytope minimizes its surface area, and vice versa.

An interesting though probably hard problem is to find (or even characterize) straight skeletons that require a minimal number of *construction events*. This question is also practically relevant, as such skeletons realize a minimum number of corners.

### Applications

Planar straight skeletons enjoy various applications, and the same can be expected for their generalizations in three dimensions. We conclude this paper with mentioning a few promising candidates.

The most direct application is *offset calculation* for triangular, or more generally, polygonal meshes. This operation is important in CAD, for example, for the thinning of solid objects and tool path generation. A suitable offset definition for the meshed surface is usually problematic. Surfaces derived from the medial axis [29] are well-defined, but they are not piecewise linear any more. Spherical and cylindrical patches have to be dealt with, which arise in the neighborhood of reflex surface components. For mitered and thus polygonal offset surfaces, many heuristics for locally repairing and filling the mesh after its facet-based or vertex-based offsetting have been proposed; see e.g. [24, 34].

Our method automatically produces a topologically correct mitered offset mesh (which can be re-triangulated if desired), and also determines the largest allowable offset threshold $\Delta$. Optimization criteria as in Sect. 10.1 can be applied, possibly combined with weighting strategies which are common in other offsetting approaches. As a byproduct, our vertex resolution process can be used to *convert* a triangulated input surface into a polygonal surface where all vertices have degree 3.

We say that a polytope ${\mathcal{Q}}$ can be *flattened* if ${\mathcal{Q}}$ can be collapsed by offsetting without tearing the surface [19]. In other words, ${\mathcal{Q}}$ can be shrunk to volume zero without the occurrence of any event that changes its topology. Clearly, ${\mathcal{Q}}$ must not contain voids or tunnels. Near-convex polytopes (Corollary 7.6) always can be flattened, because edge events do not cause topological changes, but there are also facet events with this property (see the event table in Sect. 6.5). Our straight skeleton algorithm will recognize when certain polytopes can be flattened, though we face the problem that skeletons are not unique: Whether ${\mathcal{Q}}^{\Delta }$ stays a topological ball for all $\Delta$ may depend on the shrinking process the skeleton is based on. A plausible heuristic is to resort to vertex resolution where the volume of ${\mathcal{Q}}^{\Delta }$ is maximized.

Having agreed on a particular skeleton for ${\mathcal{Q}}$, the following strategy can be used. Only edge events and facet events are performed, whose implementation is usually fast. The latter events are tested directly whether they tear the surface. Possibly arising global events are ignored, so that the obtained offset surfaces may self-intersect (a situation similar to that in Sect. 7.2). This will be detected in later edge events, though, when surface parts with a ‘wrongly’ oriented neighborhood get collapsed.

The two-dimensional case is much simpler. Exactly the near-convex polygons can be flattened, and this property can be recognized by constructing a unique straight skeleton. However, if weighting is allowed then no efficient method is known even in the planar case. A discussion of several generalized notions of convexity for polygons (being different from near-convexity) can be found in [5].

Every straight skeleton of a polytope ${\mathcal{Q}}$, and more generally every roof complex for ${\mathcal{Q}}$, defines a *decomposition* of ${\mathcal{Q}}$ into polyhedral cells. These cells are monotone in the unweighted case (Lemma 7.2), but can be of complex shape and large size. The shrinking process offers a direct means to *refine* the cells into simple shape: We can use the boundary surface of ${\mathcal{Q}}^{\Delta }$ for partitioning, after each event or in well-spaced $\Delta$-intervals. This will produce a volume mesh with node degree $\le$6 that mainly consists of prismatic cells. When the boundary facets of ${\mathcal{Q}}$ are sufficiently small (which is usually the case for boundary-triangulated polytopes obtained from approximating solid objects), then the cells in the mesh will tend to be small as well. Cells still unsuitable for a particular application, for example, in finite element methods [23], can be further refined with sheets normal to the defining facets of ${\mathcal{Q}}$, because the monotonicity property is retained in such a volume mesh. Also, cell sizes can be steered by assigning a smaller weight to facets of ${\mathcal{Q}}$ which extrude other facet’s cells. The monotonicity then may get lost, though.

We recall that a suitable roof complex *D* for the polytope ${\mathcal{Q}}$ to be volume-meshed may be computed beforehand, with the method in Sect. 7.3. Then the implementation of the shrinking process for *D* is much easier than that for any straight skeleton: The arising events only need to be processed in increasing order of time stamps, rather than also be detected, which is by far the more costly task. The time stamp $\Delta$ of an event is given by the normal distance of the respective inner corner *v* of *D* to the 4 supporting planes of ${\mathcal{Q}}$ whose $\Delta$-offsets concur in *v*.

Like in two dimensions, a straight skeleton in ${\mathbbm {R}}^3$ can attain a shape that reflects little of the distance information to the boundary of the input polytope ${\mathcal{Q}}$. This is due to reflex boundary features of ${\mathcal{Q}}$ that form small exterior angles, and may be undesirable in applications. A way to remedy this shortcoming [4], which has been systematically applied in the polygon case [30], is to ‘mitigate’ reflex vertices and edges of ${\mathcal{Q}}$ locally by introducing new facets. This can always be done, also when ${\mathcal{Q}}$ contains saddle vertices, in the offset polytope ${\mathcal{Q}}^{\Delta }$ immediately after the initial events, because ${\mathcal{Q}}^{\Delta }$ then is a simple polytope in the generic case (Sect. 6). We conjecture that a *convergence result* similar to the one in [30] can be established, showing that the skeleton of the truncated polytope approximates the medial axis of ${\mathcal{Q}}$. However, the (possibly high) arity of the initial events has to be taken into account. With a result of this kind, straight skeletons could serve as an alternative to existing piecewise-linear approximations of 3D medial axes; see e.g. [8].

## Footnotes

1 In the weighted version, the polygon edges are shifted inwards at constant but individual speeds.

2 Topological notion like boundary, interior, etc. is meant *relative* to the dimension of the object.

3 That is, the positive hull of the directed edges $\vec {vw}$ of a saddle vertex *v* is the entire ${\mathbbm {R}}^3$.

4 A subset *X* of ${\mathbbm {R}}^3$ is called *radially visible* (from the center $v \notin X$) if every ray emanating from *v* intersects *X* in at most one point. Trivially, the graph of a radial function is radially visible.

5 For two radial functions $\varphi$ and $\psi$ over the same spherical domain, their *lower* (*upper*) *envelope* is defined as the pointwise minimum (maximum) of the function values of $\varphi$ and $\psi$.

6 See Sect. 8.1 for a discussion of these issues, in the more general context of Definition 3.1.

7 Strictly speaking, *completed* should read *locally and maybe globally completed*, because sheets and cells need not be convex. We will leave out this distinction in the following.
